# Asymmetric independence modeling identifies novel gene-environment interactions

**DOI:** 10.1038/s41598-019-38983-z

**Published:** 2019-02-21

**Authors:** Guoqiang Yu, David J. Miller, Chiung-Ting Wu, Eric P. Hoffman, Chunyu Liu, David M. Herrington, Yue Wang

**Affiliations:** 1 Department of Electrical and Computer Engineering, Virginia Polytechnic Institute and State University, Arlington, VA 22203 USA; 2 Department of Electrical Engineering, The Pennsylvania State University, University Park, PA 16802 USA; 3 School of Pharmacy and Pharmaceutical Sciences, State University of New York, Binghamton, NY 13902 USA; 4 Psychiatry and Behavioral Sciences, Upstate Medical University, Syracuse, NY 13210 USA; 5 Department of Medicine, Wake Forest University, Winston-Salem, NC 27157 USA

## Abstract

Most genetic or environmental factors work together in determining complex disease risk. Detecting gene-environment interactions may allow us to elucidate novel and targetable molecular mechanisms on how environmental exposures modify genetic effects. Unfortunately, standard logistic regression (LR) assumes a convenient mathematical structure for the null hypothesis that however results in both poor detection power and type 1 error, and is also susceptible to missing factor, imperfect surrogate, and disease heterogeneity confounding effects. Here we describe a new baseline framework, the asymmetric independence model (AIM) in case-control studies, and provide mathematical proofs and simulation studies verifying its validity across a wide range of conditions. We show that AIM mathematically preserves the asymmetric nature of maintaining health versus acquiring a disease, unlike LR, and thus is more powerful and robust to detect synergistic interactions. We present examples from four clinically discrete domains where AIM identified interactions that were previously either inconsistent or recognized with less statistical certainty.

## Introduction

Detection of synergistic interaction between genetic or environmental factors aims to determine whether two or more known genetic or environmental factors jointly influence the risks of complex diseases^1–3^. Detecting such interactions is mainly driven by testing a specific biological hypothesis, and is fundamentally different from testing for association with a single factor while allowing for interaction with other factors^1,3–5^. In the context of hypothesis testing, ‘interaction’ is most commonly defined as a departure from additivity in a linear baseline model, under which these factors act independently to determine the response^1–3^. The choice of relevant statistical models may influence the accuracy and biological interpretation of inferred gene–environment interactions^1,3,6,7^.

**Figure 1:**
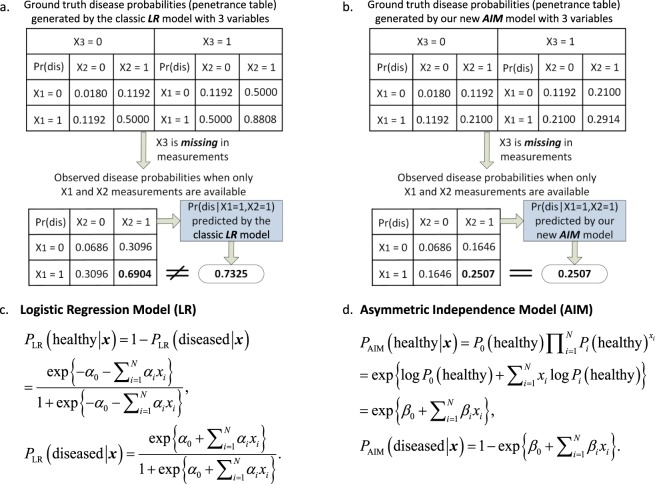
Mathematical formulation and illustrative comparison between LR and AIM. (**a**) Theoretical discrepancy between Logistic Regression (LR) prediction and ground truth probability in the case of missing variables (Appendix B). (**b**) Theoretical capability of the Asymmetric Independence Model (AIM) to accurately predict the ground truth probability in the case of missing variables. (**c**) Mathematical expression of LR. (**d**) Mathematical expression of AIM.

Interaction as a statistical concept requires the exact definition of the additive effects of the factors involved, and should always be tested together with additive effects^2,8,9^. That is, statistical interactions can only occur after additive effects have failed to explain the response, which means nothing can be established without first modelling the main effects – via a baseline independence model. Arguably, the most straightforward way to test for statistical interaction is to fit a logistic regression model (LR) with relevant interaction terms and then to test whether the interaction terms equal zero. While mathematically convenient, LR was not originated as a biological model and it is inconsistent in the presence of typically unknown confounders such as missing factors, imperfect surrogates, and disease heterogeneity (Methods and. Fig. 1a,c). Moreover, the LR model is symmetric or *exchangeable* with respect to disease status (see Methods), *i.e*. the LR mathematical form forthe probability of being healthy is the same as for the probability of being diseased. A plausible disease model, on the other hand, should be *asymmetric* with respect to disease status. In particular, one should get the disease if *any* of the risk factors are penetrant. Accordingly, being healthy requires all the risk factors to be *inactive*. Such a model is inherently asymmetric with respect to disease status (see Methods for mathematical details). A symmetric model such as LR is thus implausible as a disease model.

Thus, we address the following question: under the null hypothesis that genetic or environmental factors act independently to determine health status, how should a baseline independence model be formulated to reflect the aforementioned asymmetric nature of healthy versus diseased?

We develop an asymmetric independence model (AIM) in case-control studies for modelling the null hypothesis that attempts to mimic a sensible biological principle^10^ (Fig. 1b,d): given the independence of the marginal health status (‘healthy’ or ‘diseased’) determined probabilistically by the individual factors involved^11^, being totally ‘healthy’ requires the presence of all marginal ‘healthy’ statuses while being ‘non-healthy’ requires only at least one but not necessarily all marginal ‘diseased’ statuses. Fundamental to the success of our approach is that AIM mathematically conforms to this asymmetry by specifying being totally ‘healthy’ only if every acting factor maintains a marginal ‘healthy’ status, with the individual otherwise ‘diseased’ (Methods and Fig. 1d). Accordingly, in AIM the log-probability of being totally ‘healthy’ is linear in the factors whose coefficients correspond to the logarithms of marginal ‘healthy’ probabilities, whereas the log-probability of having disease is nonlinear in these factors (Methods and Eq. 6). Thus, a plausible disease model (AIM) is inherently an asymmetric one, unlike LR. Moreover, AIM is consistent even when the aforementioned confounders are present, both theoretically and experimentally, as seen in the sequel.

## Results

### Validation of AIM on type 1 error using simulated datasets

In the Supplement (Appendix D), we show that for all scenarios the empirical type 1 error produced by AIM closely approximates the expected type 1 error, unlike LR. We also show for AIM that the Q-Q plot closely aligns with the diagonal line with no noticeable deviation even when the factors are correlated or imbalanced.

### Comparative assessment of AIM on power of detecting interactions using simulated datasets

For power considerations, we simulated a comprehensive set of scenarios to examine how various model settings affect the performance (Appendix D and E). In most of the experiments, the ground-truth interaction models were based on an LR model with non-zero multiplicative interaction terms (Appendix D and E). The reason for this design is to assure that the LR approach is matched perfectly to the ground-truth interaction model and to show that the unsatisfactory power of LR is not in any way attributed to the interaction terms but rather is due to the LR baseline model. Note that even though AIM is not matched to the (LR) ground-truth interaction model (see Methods), AIM is guaranteed to be more powerful to detect synergistic interactions as shown in our experimental results and newly proved theorems (Supplement, Appendix C.7). Also note that when the multiplicative interaction terms are used with full parameters, this gives the same ‘saturated model’ for both LR and AIM under the alternative hypothesis (Supplement, Appendix C.7.3). Because the interaction models are under the alternative hypothesis (*e.g*., based on a logistic regression model with a non-zero interaction term), the empirical power of AIM is directly, fairly compared with that of LR. In our assessment experiments, we use the same multiplicative interaction terms to model the interaction between factors (interaction effect) in both LR and AIM under the alternative hypothesis, and then test any significant deviation of the alternative model’s likelihood from the baseline model’s likelihood.

**Figure 2:**
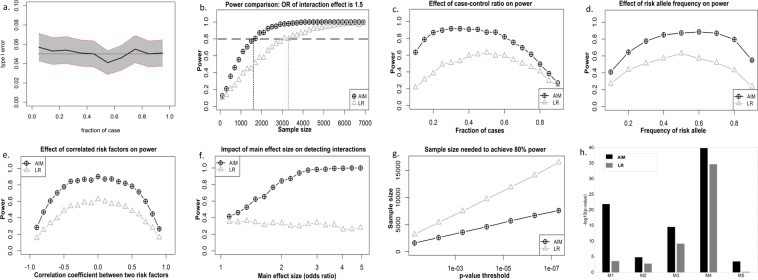
Comparative performance assessment of AIM and LR using extensive simulation datasets. Our extensive simulation studies evaluate the type 1 error and detection power of AIM and LR in a controlled setting, under varying parameter settings which characterize the population being studied, as well as under the three confounding scenarios prominently identified in this paper – missing factors, surrogate factors, and disease subtypes. The goal is to understand the performance effects of different parameter settings and of these scenarios on both models. (**a**) The empirical type I error (evaluated when the null hypothesis of no interaction is valid) at significance level 0.05. The gray region is the 95% confidence interval. (**b**) Power versus sample size with interaction effect size at an odds ratio of 1.5; and case fraction of 50% and the main effect size of 1.5 for both risk factors. (**c**) Power versus case-control ratio. The fraction of cases is varied by adjusting the baseline parameter in the LR model possessing an interaction term. The sample size is 2000 and the interaction effect size is 1.5. The main effect size for both risk factors is 1.5. (**d**) Power versus frequency of risk allele, with sample size 2000, main effect size 1.5 for both risk factors, interaction effect size 1.5, and case fraction at 50%. (**e**) Power to detect an interaction versus correlation between the risk factors for AIM and LR models; both methods achieve their greatest detection power when risk factors are uncorrelated. (**f**) Power versus main effect size, with sample size 1000, interaction effect size 1.5, and case fraction 50%. (**g**) Sample size versus p-value threshold, with main effect size 1.5, interaction effect size 1.5, and case fraction 50%. (**h**) Statistical significance (log p-values) of five ground-truth interactions, as detected by the AIM and LR models (Appendix D–E).

Experimental results for different sample sizes show that AIM consistently exhibits higher power than LR; that is, to achieve the same power, AIM requires much fewer samples compared to LR (Fig. 2b). The relatively larger gain by AIM for smaller effect sizes with limited samples, which often occurs in real applications, is particularly beneficial (Supplementary Fig. S6a–c). Experimental results also show that AIM consistently produces higher power than LR with varying case-control ratio (Fig. 2c), allele frequency (Fig. 2d), and factor correlation (Fig. 2e). Concerning the impact of main effect size (additive portion) (Fig. 2f), we notice that AIM’s power quickly increases while LR’s power slightly decreases as the main effect size increases. These divergent trends may be expected because an interaction becomes more obvious when the main effect is accurately estimated by AIM. Moreover, it is practically advantageous that, to achieve both high sensitivity and specificity, AIM needs about half of the sample size required by LR (Fig. 2g). We again emphasize that, in all of these comparisons, the same (1000) data set realizations, based on a ground-truth LR model with interaction terms, were used to assess power for both LR and AIM. Thus, there is a fair comparative assessment of power between AIM and LR.

We also tested AIM on existing simulation data derived from real single nucleotide polymorphism (SNP) study data, as part of the New York City Cancer Control Project. This data set was used in previous studies on interaction detection in genome-wide association studies^12,13^. The data set includes sub-populations that possess one (or more) distinct interactions, with five interactions in total. The interaction models vary in the order of the interaction (up to 5-way interactions), genetic models, incomplete/complete penetrance, minor allele frequency, and marginal effects size. The interaction models jointly determine the disease status for each individual; thus, the disease status in this data set is generated in a fashion quite different from both the LR and AIM interaction models. Full details on this data set can be found in the literature^12^. Again, superior power of AIM is observed for this data set (Fig. 2h).

### Comparative assessment of AIM in the presence of confounders using simulated datasets

**Figure 3:**
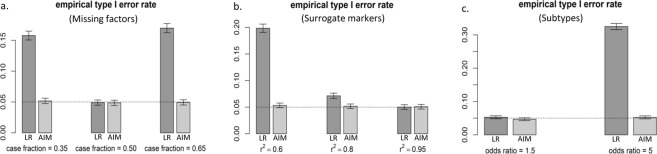
Empirical type I error rate at significance level 0.05 for LR (dark grey) and AIM (light grey). (**a**) A few missing factors with large effect size; (**b**) Surrogate markers with strong marginal effects; (**c**) Three subtypes.

Specificity in detecting interactions can be greatly hampered by missing factors, imperfect surrogates, and disease heterogeneity, where ‘interaction’ is most commonly defined as a departure from additivity in a linear baseline model in which these (‘imperfect’) factors act independently to determine the response (Fig. 1a,b). We investigated the impact of such confounders on the type 1 error both theoretically (Methods) and experimentally (Supplementary Fig. S4). Using extensive simulations with various model parameter combinations, we show that for all scenarios AIM maintains accurate and robust empirical type 1 error rates that match almost perfectly the theoretical significance level, in the presence of missing factors (Fig. 3a**)**, imperfect surrogates (Fig. 3b), and disease heterogeneity (Fig. 3c). In contrast, for the same experimental settings LR produces inflated type 1 error rates (Fig. 3a–c) attributable to its mathematical inconsistency **(**Appendix B), resulting in more unwanted false positives specifically with larger main effect sizes.

### Application of AIM on real venous thrombosis dataset detects interaction between variants of factor V and prothrombin contributing to increased risk of venous thrombosis

As an example of gene-environment interaction, the synergistic influence of thrombophilic mutation (R506Q and G20210A) and oral contraceptive on venous thrombosis is well-established by multiple epidemiological studies (Table 1), with an observed odds ratio of 27.4 compared to the additive effect odds ratio of 9.34^14,15^. Mechanistically, R506Q substitution in factor V involves one of three sites that are cleaved by activated protein C, resulting in augmented generation of thrombin; and G20210A mutation in the 3′ untranslated region of the prothrombin gene is associated with producing thrombin and activating factor Va^16^. In addition, oral contraceptives have long been recognized as a risk factor for venous thrombosis, with significant effect on producing thrombin via decreasing factor V and increasing prothrombin. Our AIM analysis of this case confirms the synergistic interaction with a p-value of 6.2e-4, much more confidently than the p-value of 0.021 assessed by LR. This result confirms not only the previously reported synergistic interaction but also AIM’s ability to detect it correctly and surely (Methods).

**Table 1:** Legnani *et al*. study: risk of venous thrombosis according to the presence of thrombophilic genetic mutation and the use of oral contraceptive.

Thrombophilic genetic risk mutation	Oral contraceptive	Controls	Cases	Odds ratio
−	−	444	118	1
−	+	166	86	1.95
+	−	33	42	4.79
+	+	7	51	27.4

### Application of AIM on real esophageal cancer dataset detects smoking-alcohol interaction contributing to increased risk of esophageal cancer

Epidemiological studies have shown the synergistic interplay of tobacco smoking and alcohol consumption on various cancers. Specifically, studies have shown that the combination of the two factors significantly increased esophageal cancer risk more than either of them separately, where alcohol may act as a cocarcinogen that enhances the carcinogenic effects of tobacco smoking^17,18^. However, the previously reported findings were inconsistent in that the evidence was significant in women and in all subjects but not in men (Table 2)^18,19^. Separately analyzing the groups of men, women, and all (Methods), AIM produces consistent evidence across these groups with p-values of 5.43e-6, 3.1e-3, and 2.11e-8, respectively. On the same dataset, contradictory results remain for LR (Methods).

**Table 2:** Joint association of alcohol drinking and tobacco smoking statuses with esophageal cancer risk.

Alcohol	Smoking	Men			Women			All		
		Control	Case	Odds ratio	Control	Case	Odds ratio	Control	Case	Odds ratio
never	never	189	8	1	234	83	1	423	91	1
never	ever	298	61	4.84	55	27	1.38	353	88	1.16
ever	never	144	24	3.94	63	29	1.30	207	53	1.19
ever	ever	777	562	17.1	19	36	5.34	796	598	3.49
LR (*p*)		0.81			0.014			5.10e-5		
AIM (*p*)		5.43e-6			0.0031			2.11e-8		

### Application of AIM on real esophageal cancer dataset detects ALDH2-alcohol interaction contributing to increased risk of esophageal cancer

**Figure 4:**
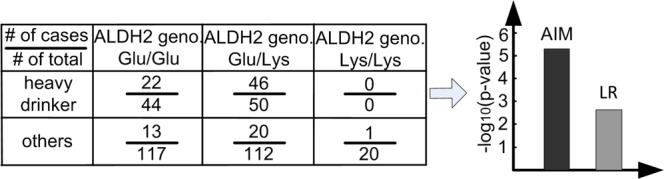
Re-analysis of the interaction between the ALDH2 gene and alcohol consumption.

Both the ALDH2 gene and alcohol consumption are known risk factors associated with esophageal cancer. Heavy alcohol consumption has been found to be a risk factor for esophageal cancer in many epidemiological studies^20^. When alcohol is metabolized in the liver, it is broken down to acetaldehyde, a carcinogen that binds to cellular protein and DNA. The ALDH2 protein is responsible for degrading the carcinogen, and a functional polymorphism in the ALDH2 gene significantly reduces such capacity^21^. We re-analyzed the data of ALDH2-alcohol interaction effect on esophageal cancer to reinterpret marginally significant ALDH2 and alcohol consumption on the basis of their synergistic effects (Fig. 4). The significance assessed by AIM produces a p-value of 7.4e-6, compared to a p-value of 2.5e-3 with LR, an almost thousand-fold improvement (Methods).

### Application of AIM on real bladder cancer dataset detects NAT2-smoking interaction contributing to increased risk of bladder cancer

Multiple carcinogens have been found in tobacco smoke, and these carcinogens may undergo both activation and de-toxification. The NAT2 gene encodes an enzyme that functions to both activate and deactivate arylamine and hydrazine carcinogens. The association of the NAT2 slow acetylator with bladder risk, caused by the polymorphisms in the NAT2 gene, is quite well established^22^. We re-analyzed this bladder cancer dataset to confirm the NAT2-smoking interaction. The significance assessed by AIM produces a p-value of 0.0011, compared to a p-value of 0.015 with LR (Table 3). Multiple previous studies have consistently shown the interaction between the NAT2 gene and smoking on bladder cancer, where such interaction is evident because the observed odds ratio is 2.89 while the odds ratio in the presence of both factors is predicted to be 1.69 by the multiplicative model (Methods).

**Table 3:** Joint association of tobacco smoking status and NAT2 acetylation genotype with bladder cancer risk.

NAT2 acetylation genotype	Smoking status	Controls	Cases	Odds ratio
Fast	never	131	66	1
Fast	ever	362	340	1.86
Slow	never	199	91	0.91
Slow	ever	438	637	2.89

## Discussion

Detecting synergistic interactions among risk factors is a fundamental task in clinical and population research. Few previous studies have addressed the problem of detecting interaction among known genetic or environmental factors^3^, and without exception, they adopt the LR framework^3–5^. However, while hypothesis testing using LR with interaction terms is a convenient solution and is widely used in practice, the LR framework is poorly powered and ill-suited under several commonly occurring circumstances, including missing or unmeasured risk factors, imperfectly correlated surrogates, and multiple disease sub-types. The weakness of LR in these settings stems from the way the null hypothesis is defined (Appendix B).

In this report we propose the AIM framework as a biologically-inspired alternative to LR, based on the key observation that the mechanisms associated with acquiring a “disease” versus maintaining “health” are asymmetric. We have shown that AIM analysis on benchmark real datasets not only more confidently confirms known interactions but also successfully reconciles inconsistent interactions. Across all of our real data set experiments, AIM demonstrated enhanced power compared to LR. We further checked the types of interactions and found that they are all synergistic – in all of these applications, carrying double risk factors engendered larger risk than expected based just on additive effects. Supported theoretically by newly proved theorems and experimentally by comprehensive simulation studies, we conclude that the extra power and robust specificity gained by AIM relative to that of LR is attributable to two properties rooted in the AIM formulation: its asymmetry and mathematical consistency. To the best of our knowledge, AIM represents the first model that mathematically preserves the asymmetry between being totally ‘healthy’ and ‘non-healthy’^10^ and explicitly relates its model coefficients to marginal ‘healthy’ probabilities. As a result, AIM guarantees a larger likelihood difference for synergistic interactions under alternative versus null hypotheses than that of LR (Appendix C–E).

## Methods

### LR overview

Baseline LR posits a log-linear odds in terms of the posterior probability on healthy/diseased status, *i.e*.,

1 $$\mathrm{log}\,\frac{{P}_{{\rm{LR}}}({\rm{diseased}}|{\boldsymbol{x}})}{{P}_{{\rm{LR}}}({\rm{healthy}}|{\boldsymbol{x}})}={\alpha }_{0}+{\sum }_{i=1}^{N}{\alpha }_{i}{x}_{i},$$

where ***x*** is the vector of *N* binary health status variables, and ***α*** is the vector of regression coefficients. In our discussion, ‘*x*_*i*_ = 1’ means that the *i*th disease factor is *active*, and ‘*x*_*i*_ = 0’ means that the *i*th disease factor is *inactive*. By some simple mathematical manipulations, LR can also be expressed as

2 $${P}_{{\rm{LR}}}(\mathrm{diseased}|{\boldsymbol{x}})=\frac{\exp \{{\alpha }_{0}+{\sum }_{i=1}^{N}{\alpha }_{i}{x}_{i}\}}{1+\exp \{{\alpha }_{0}+{\sum }_{i=1}^{N}{\alpha }_{i}{x}_{i}\}},$$

3 $${P}_{{\rm{LR}}}(\mathrm{healthy}|{\boldsymbol{x}})=1-{P}_{{\rm{LR}}}(\mathrm{diseased}|{\boldsymbol{x}})=\frac{\exp \{-{\alpha }_{0}-{\sum }_{i=1}^{N}{\alpha }_{i}{x}_{i}\}}{1+\exp \{-{\alpha }_{0}-{\sum }_{i=1}^{N}{\alpha }_{i}{x}_{i}\}}.$$

Because LR is adopted mainly for mathematical convenience but not biological plausibility, the vital and statistical relationship between the marginal ${P}_{{\rm{LR}}}(\mathrm{healthy}|{x}_{i})$ and the overall ${P}_{{\rm{LR}}}(\mathrm{healthy}|{\boldsymbol{x}})$ probabilities on health status is largely lost.

### LR limitations

Note that (2) and (3) have the same form, *i.e*. LR is symmetric with respect to disease status. This symmetric form is not biologically plausible considering causality of diseases. Specifically, a common concept is that one may get the disease if any one of the risk factors are penetrant or active, whereas being healthy requires all of the factors to be inactive. This conceptual model is inherently asymmetric with respect to the two health statuses, diseased and healthy. In contrast, LR makes no distinction in mathematically defining diseased or healthy subjects.

Moreover, LR is invalid in the presence of many common confounders in practice. Because the prevailing scenario regarding complex diseases is that we often have incomplete knowledge of the true risk factors, the major confounders include missing/unmeasured factors and imperfect surrogates. We have shown that the LR parametric form is not invariant to these two effects and there is no way to “correct” LR for these potentially confounding effects in practice. For example, suppose there are three binary causal factors; when all three factors are observed we have model LR-3; Suppose now that the third risk factor is missing. If LR is invariant to missing factors, then marginalizing out the third risk factor from LR-3 should yield a model with the LR parametric form based on the two remaining risk factors. However, it is shown that the marginalized model does not have the LR parametric form (Fig. 1c and Appendix B). In a similar fashion, also by counterexample, we have shown that the prediction of health status by LR is not invariant to imperfect surrogates. In conclusion, in the presence of these common confounders, LR is theoretically biased which, as will be shown experimentally in this report, results in either inflated type 1 error or reduced power or both (Appendix D–E).

### Asymmetric independence model

In developing the AIM null hypothesis model, we assume that risk factors independently exert effects on health status, expressed mathematically as

4 $$P(c|{\boldsymbol{x}})={\prod }_{i=1}^{N}P({c}_{i}|{{\boldsymbol{x}}}_{i}),$$

where ${c}_{i}\in \{0/{\rm{healthy}},1/{\rm{diseased}}\}$ is the latent ‘local’ disease status random variable coupled to each factor ${x}_{i}$, *i.e*., with the ${c}_{i}$ assumed statistically independent of each other given the status of ${x}_{i}$. We also assume that the factor being active is required for the local status to be ‘diseased’, *i.e*., $P({c}_{i}=1|{x}_{i}=0)=0$; on the other hand, the active factor probabilistically causes the local status to be “diseased” based on the conditional probability ${\varphi }_{i}=P({c}_{i}=1|{x}_{i}=1)$. As one example, in one of the two esophageal cancer studies, there are two binary factors, ${x}_{1}$ and ${x}_{2}$_,_ representing presence/absence of smoking and alcohol consumption, respectively. Each of these factors is coupled to a local disease status variable, ${c}_{i}$, $i=1,2$. The probability $P[{c}_{1}=1\,|{x}_{1}=1]$ is the propensity for disease (${c}_{1}=1$) given that an individual is a smoker. Likewise, there is a propensity for disease given that the individual is an alcohol consumer, $P[{c}_{2}=1|{x}_{2}=1]$. We further assume that an overall healthy status occurs only if every *active* factor does not cause its local status to be ‘diseased’, expressed mathematically as

5 $$P(c=0|{\boldsymbol{x}})=P({c}_{0}=0){\prod }_{i=1}^{N}P({c}_{i}=0|{x}_{i}),$$

where ${c}_{0}$ is a ‘background’ status accounting for sporadic disease occurrence that cannot be explained by any active factor, with probability ${\varphi }_{0}=P({c}_{0}=1)$. Then, AIM can be expressed as

6 $$\begin{array}{ccc}{P}_{{\rm{AIM}}}(\mathrm{healthy}|{\boldsymbol{x}}) & = & {P}_{{\rm{0}}}({\rm{healthy}}){\prod }_{i=1}^{N}{P}_{i}{({\rm{healthy}})}^{{x}_{i}}\\  & = & \exp \{\mathrm{log}(1-{\varphi }_{0})+{\sum }_{i=1}^{N}{x}_{i}\,\mathrm{log}(1-{\varphi }_{i})\}\\  & = & \exp \{{\beta }_{0}+{\sum }_{i=1}^{N}{\beta }_{i}{x}_{i}\},\end{array}$$

7 $${P}_{{\rm{AIM}}}(\mathrm{diseased}|{\boldsymbol{x}})=1-\exp \{{\beta }_{0}+{\sum }_{i=1}^{N}{\beta }_{i}{x}_{i}\},$$

where the regression coefficient can be explicitly interpreted as the logarithm of the local healthy probability, *i.e*., ${\beta }_{i}=\,\mathrm{log}[1-P({c}_{i}=1|{x}_{i}=1)]=\,\mathrm{log}\,P({c}_{i}=0|{x}_{i}=1)$.

Because mechanisms of being healthy and diseased are different, in contrast to LR, AIM is specifically formulated to be asymmetric with respect to disease status, with the log-probability of being healthy a linear function of the factors (6) whereas the log-probability of being diseased is clearly nonlinear (7). Furthermore, AIM is supported by several well-accepted biological models, including the heterogeneity theory^10^ and the two-hits theory of cancer^11^ (Appendix C.4). While we have argued that AIM is more biologically plausible than LR, we believe the most compelling support for AIM comes from the *invariance* of this model, unlike LR, in the presence of common confounders such as missing factors, imperfect surrogates, and disease heterogeneity. We emphasize that *no* modifications of the model given in (6) and (7) are needed to achieve AIM’s invariance to these confounders. The mathematical proofs of AIM’s invariance to these common confounders are given in (Appendix C.5–7). We also point out that, similar to the logistic regression model, AIM can readily account for covariate effects, if observed, by including extra terms corresponding to these covariate factors. Lastly, we have shown that maximum likelihood estimation of the AIM model is a convex optimization problem and we have developed an efficient learning algorithm (Appendix C.2–3).

### Likelihood function for AIM

Consider a case-control population $${\boldsymbol{X}}=\{({{\bf{x}}}_{i},{{\bf{I}}}_{i}),\,i=1,\ldots ,M\}$$ where $${{\bf{x}}}_{i}$$ is the factor vector for the $$i$$-th subject and $${{\bf{I}}}_{i}\,=\,1$$ for a case and $${{\bf{I}}}_{i}\,=0$$ for a control. Let $${{\bf{y}}}_{i}={[1{{\bf{x}}}_{i}]}^{{\rm{T}}}$$ and $${\bf{b}}=[{b}_{0},{b}_{1}$$$$,\ldots ,{b}_{N}]$$. The likelihood of ***X*** under the AIM model is:

$${P}_{AIM}[{\boldsymbol{X}}]\,=\,\prod _{\{i=1\}}^{M}{P}_{AIM}{[C=1|{{\bf{x}}}_{i}]}^{{{\rm{I}}}_{i}}{P}_{AIM}{[C=0|{{\bf{x}}}_{i}]}^{1-{{\rm{I}}}_{i}}$$, with the log-likelihood given by: $$L({\boldsymbol{b}})\equiv \,\mathrm{log}$$$$({P}_{AIM}[{\boldsymbol{X}};{\bf{b}}])=\,\sum _{i=1}^{M}((1\,\mbox{--}\,{{\bf{I}}}_{i}){{\bf{b}}}^{{\boldsymbol{T}}}{{\bf{y}}}_{i}+{{\bf{I}}}_{i}\,\mathrm{log}(1\,\mbox{--}\,{e}^{{{\bf{b}}}^{{\boldsymbol{T}}}{{\bf{y}}}_{i}}\,))$$.

This is a convex function of the parameter vector ${\bf{b}}$ (Appendix C.2) with the resulting maximum likelihood estimation (MLE) learning problem a convex optimization problem, amenable to finding the global maximum.

### Likelihood ratio test for AIM

Given a case-control population ***X****,* one performs MLE to learn the AIM null hypothesis model (no interaction), with log-likelihood $\mathrm{log}({P}_{AIM}[{\boldsymbol{X}};{{\bf{b}}}_{{\boldsymbol{null}}}])$. To test for an interaction between factors ${x}_{i}$ and ${x}_{j}$ one adds an interaction term of the form ${\beta }_{ij}{x}_{i}{x}_{j}$ to the AIM posterior in equations (6) and (7) and MLE-learns the AIM alternative posterior, with parameter vector ${{\bf{b}}}_{alt}$ and log-likelihood $\mathrm{log}({P}_{AIM}[{\boldsymbol{X}};{{\bf{b}}}_{alt}])$. A standard log-likelihood ratio test (the same one applied for LR) is then applied to $2(\mathrm{log}({P}_{AIM}[{\boldsymbol{X}};{{\bf{b}}}_{alt}])-\,\mathrm{log}({P}_{AIM}[{\boldsymbol{X}};{{\bf{b}}}_{null}]))$ since the AIM log-likelihood ratio is asymptotically chi-squared.

### Evaluation of type 1 error

Extensive experiments evaluating type 1 error for AIM and LR are found in the Supplementary Information.

### Theoretical Characterization of Interaction Detection Power for AIM and LR

Extensive experiments evaluating detection power for AIM and LR are found in the Supplementary Information, with a theoretical proof of AIM’s superior power given in Appendix C.7.

### Detecting interaction in venous thrombosis dataset

The interaction between thrombophilic mutations and oral contraceptive is well-established, with multiple epidemiological and mechanical studies^14,15,23,24^. In the Legnani *et al*. study, the odds ratio associated with the use of oral contraceptive but no thrombophilic genetic risk mutation is 1.95, and the odds ratio associated with genetic defects but no use of contraceptive is 4.79. There is strong evidence of interaction. Indeed, by applying LR, we get a p-value of 0.021, which is statistically significant. There are 947 subjects in the Legnani *et al*. study. When all the frequencies of the risk factors and the effect size are kept the same, we estimate that, to achieve the 0.05 significance level, LR requires 676 subjects, while AIM needs only 303 subjects. For the Martinelli *et al*. study, the odds ratio associated with the presence of both risk factors is expected to be 11.9, compared to the observed value of 18.1. Both studies have the same effect direction, that is, the observed odds ratio is larger than the expectation. Due to the limited sample size, the conclusion is not statistically significant in the Martinelli *et al*. study. The p-value generated by LR is 0.618 and the p-value obtained from AIM is 0.183. To achieve the 0.05 significance level, the estimated sample size associated with LR is 4391, while AIM requires just 614 subjects.

### Detecting smoking-alcohol interaction in esophageal cancer dataset

The data are divided into three groups – males, females, and all subjects. In each group, we calculate the interaction effect based on LR and AIM. We can see that the new model consistently generates smaller p-values than LR. In the males group, the p-value is 5.43e-6 based on the new model, while it is 0.81 for LR and far from being considered significant. We also estimate the sample sizes required for the two models to achieve the 0.05 significance level, again assuming that all the frequencies of the risk factors and the effect size are kept the same. In the males group, LR needs 131413 subjects, compared to just 374 subjects required for AIM. In the females group, LR needs 339 subjects and AIM needs 235. In the all group, 596 subjects are necessary for LR, while 312 subjects are sufficient for AIM.

### Detecting ALDH2-alcohol interaction in esophageal cancer dataset

The data were collected from the first study of the ALDH2-alcohol interaction effect on esophageal cancer. The original report discovered the interaction effect via LR, which was confirmed by follow up studies to be a true interaction^21^. The distribution of the cases and the controls are presented in Fig. 4. There are in total 343 subjects in the study. When all the frequencies of the risk factors and the effect size are kept the same, we estimate that, to achieve the 0.05 significance level, LR requires 142 subjects while AIM needs only 64 subjects.

### Detecting NAT2-smoking interaction in bladder cancer dataset

Multiple studies have consistently shown the interaction between the NAT2 gene and smoking on bladder cancer. Table 3 presents the non-meta-analysis study with the largest sample size. Choosing the bladder cancer risk for “never smoked” and NAT2 fast acetylator as the reference, the odds ratio associated with “smoked before” (i.e., an individual who has smoked before) and NAT2 fast acetylator is 1.86, and the odds ratio associated with “never smoked” and NAT2 slow acetylator is 0.91. According to the multiplicative model, the odds ratio associated with the presence of both risk factors should be 1.69, while the observed odds ratio is 2.89. So an interaction is evident. There are 2264 subjects in the study. When all the frequencies of the risk factors and the effect size are kept the same, we estimate that, to achieve the 0.05 significance level, LR requires 1449 subjects and AIM needs 796 subjects.

## Supplementary information

Supplementary file
