# Supplementary material for: Asymmetric independence modeling identifies novel gene-environment interactions
Source: Sci Rep. 2019 Feb 21;9:2455. doi: 10.1038/s41598-019-38983-z (PMC6385186; doi:10.1038/s41598-019-38983-z)
Supplement: Supplementary file 1 — Supplementary file [file 41598_2019_38983_MOESM1_ESM.pdf]

Suppl. Information: Asymmetric Independence Modeling identifies novel gene-environment interactions

Guoqiang Yu*
Dept. of Electrical and Computer Engineering, Virginia Polytechnic Institute and State University, Arlington, VA, USA.

David J. Miller
Dept. of Electrical Engineering, The Pennsylvania State University, University Park, PA, USA.

Chiung-Ting Wu
Dept. of Electrical and Computer Engineering, Virginia Polytechnic Institute and State University, Arlington, VA, USA.

Eric P. Hoffman
School of Pharmacy and Pharmaceutical Sciences, State University of New York, Binghamton, NY, USA

Chunyu Liu
Psychiatry and Behavioral Sciences, Upstate Medical University, Syracuse, NY, USA

David M. Herrington
Dept. of Medicine, Wake Forest University, Winston-Salem, NC, USA.

Yue Wang
Dept. of Electrical and Computer Engineering, Virginia Polytechnic Institute and State University, Arlington, VA, USA.

Appendix A: Novel Contributions of The Paper

Detection of interactions is an old problem and there is quite a large body of literature on this topic, as we elaborated on in the main article. AIM differs principally from the two main standard models in the field, *i.e.*, the aforementioned multiplicative and additive models. AIM is based on the principle of independent effects between causes. Similar principles have been explored in the literature, particularly in epidemiology, and variants of our AIM model have been proposed under simplifying assumptions such as rare occurrence of diseases and no covariates[1–3]. However, no previous effort has exploited the principle in its complete and authentic form, as AIM does here. More importantly, this paper makes significant efforts to comprehensively characterize the

*to whom correspondence should be addressed

properties of the derived model and their practical consequences, which is much needed to deepen understanding and guide applications to real problems. Situating AIM relative to the current literature, we make a number of novel contributions as follows: (1) We build a stochastic (null) model (AIM) based on the principle of independent causal effects. This model can naturally account for covariates, handle non-binary factors, as well as more than two factors. (2) We prove the convexity of AIM's maximum likelihood problem and apply convex optimization theory to estimate the parameters. (3) We prove the consistency of AIM when missing or unmeasured causal factors exist. (4) We prove the consistency of AIM when imperfectly correlated surrogate markers are measured, rather than the true causal factors. (5) We prove the consistency of AIM when there is disease heterogeneity or subtypes. (6) For interaction detection, we make a theoretical power comparison between AIM and logistic regression (LR) and identify (quite practical) conditions under which AIM is guaranteed to have larger power than LR (as well as conditions under which LR has larger power than AIM). (7) Through simulation studies, we extensively survey the effect of various factors, such as case fraction, main effect size and correlation between variables, on both type I error and power of AIM and LR. Among these contributions, some are shared by efforts on other models. For example, it is easy to account for covariates under LR[4]. Consistency under missing or unmeasured causal factors has been discussed for both multiplicative and additive models[5]. It has also been demonstrated that the additive model is consistent under measurement errors or misclassifications, which is closely related to the scenario of surrogate markers[6]. However, to the best of our knowledge, all these contributions listed above are new for models using the principle of independent causal effects. Most notably, we are the first to characterize the power difference between models in a mathematically rigorous way and to identify precise conditions under which one is better powered than the other.

Appendix B: Model Inconsistency of LR

B.1 Limitations of LR

In general, the "correct" null form is both unknown and domain-dependent. Thus, in choosing a model one must be guided by several desiderata: 1) theoretical plausibility – is the model derivable starting from plausible assumptions and domain knowledge ?; 2) model parsimony; 3) model *robustness* in the face of commonly occurring confounding effects; 4) experimental support/validation. Several such considerations are not well met by LR.

B.2 LR framework is (in some instances) biologically implausible

LR is not particularly inspired by nor derivable from an underlying conceptual model of disease risk. Indeed, it was originally proposed to model population growth[7]. One feature of LR that is inconsistent with a plausible disease model is its symmetry or exchangeability with respect to the class label status. Specifically, when modeling the relationship between two or more risk factors and a binary disease outcome, a plausible conceptual model is that one gets the disease if any of the risk factors are penetrant – accordingly, being healthy requires all of the factors to be inactive. Such a model is inherently *asymmetric* with respect to the two statuses – diseased and healthy. However, in LR, the two statuses are exchangeable and the model is thus symmetric, i.e. $\log(P_{LR}(C = 1|\underline{X})/P_{LR}(C = 0|\underline{X}) = \sum_{i=1}^{N} \alpha_i X_i + \alpha_0$, and if we swap disease and health labels, that is, $C = 1 \Leftrightarrow C' = 0$ and $C = 0 \Leftrightarrow C' = 1$, we have $\log(P_{LR}(C' = 1|\underline{X})/P_{LR}(C' = 0|\underline{X}) = \sum_{i=1}^{N}(-\alpha_i)X_i + (-\alpha_0)$. That is, the LR form is invariant to label swapping; moreover, the data likelihood, with estimated parameters plugged in, is also invariant to label swapping. That is, for LR it does not matter whether the case group is defined to consist of diseased or healthy subjects. Equivalently, under LR, one does not require all the risk factors to be inactive in order to be healthy. Even without more detailed consideration, LR, as a symmetric model, conflicts with a common conceptual model of disease risk.

B.3 An example of the missing factors

As the example in the result section, suppose there are three binary causal factors ($N = 3$) and that the LR baseline joint effects model, when all three binary causal factors are observed, has parameter values: $\alpha_0 = -4$, $\alpha_1 = 2$, $\alpha_2 = 2$, and $\alpha_3 = 2$. The upper table in Figure 1c shows the disease probability under each of the eight combinations. Suppose now that the third risk factor X_3 is not observed (and, in fact, is not known to exist). Further, suppose that $P[X_3 = 0] = P[X_3 = 1] = 0.5$. Then the distribution of the disease probability for this case (with X_3 unobserved) is obtained by averaging the left and right sub-tables of the upper table in Figure 1c. The new distribution is shown in the lower table in Figure 1c [Note, however, that this table cannot be computed in practice, because in this confounding scenario, X_3 is not only unobserved – it is not *known* to be a disease factor]. Assuming that the LR model form is preserved when X_3 is not observed, let us denote by α'_0, α'_1, and α'_2 the parameter values for the new LR model. As there are three parameters, any three combinations from the four combinations in the lower table in Figure 1c

suffice to calculate the parameters. We choose to compute these by excluding $(X_1 = 1, X_2 = 1)$, as follows:

$$\alpha'_0 = \log\left(\frac{P(C = 1|X_1 = 0, X_2 = 0)}{1 - P(C = 1|X_1 = 0, X_2 = 0)}\right) = \log\left(\frac{0.0686}{1 - 0.0686}\right) = -2.6084$$

$$\alpha'_1 = \log\left(\frac{P(C = 1|X_1 = 0, X_2 = 1)}{1 - P(C = 1|X_1 = 0, X_2 = 1)}\right) - \alpha'_0 = \log\left(\frac{0.3096}{1 - 0.3096}\right) + 0.0711 = 1.8064$$

$$\alpha'_2 = \log\left(\frac{P(C = 1|X_1 = 1, X_2 = 0)}{1 - P(C = 1|X_1 = 1, X_2 = 0)}\right) - \alpha'_0 = \log\left(\frac{0.0686}{1 - 0.0686}\right) + 0.0711 = 1.8064$$

The odds for the combination $(X_1 = 1, X_2 = 1)$ based on the LR model is:

$$\frac{P(C = 1|X_1 = 1, X_2 = 1)}{1 - P(C = 1|X_1 = 1, X_2 = 1)} = e^{\alpha'_0 + 2\alpha'_1} = 2.7385. \tag{1}$$

However, from the lower table in Figure 1c, the odds for $(X_1 = 1, X_2 = 1)$ is $\frac{0.6904}{1-0.6904} = 2.2300$. Thus, the assumption that the LR form is preserved when there are missing factors (*i.e.*, estimating new LR parameter values only for the observed factors is equivalent to marginalizing over the missing factors) is contradicted in this example and, thus, as a general rule.

In a similar fashion, also by counterexample, one can show that the LR form is *also* not invariant to observation of *surrogate* factors, correlated with the true factors, rather than the true ones. That is, properly accounting for the correlation structure between measured surrogates and their "upstream" causal factors does not preserve the LR model parametric form.

B.4 An example of the presence of surrogate factors

Consider an example that there are two binary causal factors ($N = 2$) and where, when both causal factors are observed, the baseline LR model has parameters $\alpha_0 = -4$, $\alpha_1 = 2$, and $\alpha_2 = 2$. The corresponding disease distribution is shown in Table S1.

However, suppose now that, rather than observing the causal factors X_1 and X_2, we observe two surrogate factors, X'_1 and X'_2, correlated with their respective causal factors and statistically independent of each other. Assume $P(X'_m = 0|X_m = 0) = 0.9$, $P(X'_m = 1|X_m = 1) = 0.9$, and let $P(X_m = 0) = 0.5, m = 1, 2$.

p	X2 = 0	X2 = 1
X1 = 0	0.0180	0.1192
X1 = 1	0.1192	0. 5000

Table S1. Posterior probability of disease with two causal SNPs X_1 and X_2 under the logistic regression model.

Applying Bayes rule and using the fact, for this example, that $P(X_1 = i, X_2 = j) = P(X_1' = i', X_2' = j') = 0.25, \forall i, j, i', j'$, we find that

$$\begin{aligned} & P_{LR}(C = 1|X_1' = k_1, X_2' = k_2) \quad (2) \\ = & \sum_{i=0}^{1} \sum_{j=0}^{1} P_{LR}(C = 1|X_1 = i, X_2 = j) P(X_1' = k_1, X_2' = k_2|X_1 = i, X_2 = j). \end{aligned}$$

The resulting disease distribution is shown in Table S2.

p	X2' = 0	X2' = 1
X1' = 0	0.0410	0.1444
X1' = 1	0.1444	0.4266

Table S2. Posterior probability of disease with two surrogate variables X_1' and X_2' under the logistic regression model.

Now, assuming that the LR model form is preserved when the surrogate factors, rather than the causal factors, are observed, let us denote by α_0', α_1', and α_2' the parameter values for the new LR model. We can compute these based on Table S2, as follows:

$$\begin{aligned} \alpha_0' &= \log\left(\frac{P(C = 1|X_1' = 0, X_2' = 0)}{1 - P(C = 1|X_1' = 0, X_2' = 0)}\right) = \log\left(\frac{0.0410}{1 - 0.0410}\right) = -3.1523 \\ \alpha_1' &= \log\left(\frac{P(C = 1|X_1' = 1, X_2' = 0)}{1 - P(C = 1|X_1' = 1, X_2' = 0)}\right) - \alpha_0' = \log\left(\frac{0.1444}{1 - 0.1444}\right) + 3.1523 = 1.3731 \\ \alpha_2' &= \log\left(\frac{P(C = 1|X_1' = 0, X_2' = 1)}{1 - P(C = 1|X_1' = 0, X_2' = 1)}\right) - \alpha_0' = \log\left(\frac{0.1444}{1 - 0.1444}\right) + 3.1523 = 1.3731 \end{aligned}$$

The odds for the combination $(X_1' = 1, X_2' = 1)$ based on this model is:

$$\frac{P(C = 1|X_1' = 1, X_2' = 1)}{1 - P(C = 1|X_1' = 1, X_2' = 1)} = e^{\alpha_0' + \alpha_1' + \alpha_2'} = 0.6662. \quad (3)$$

However, from Table S2, the odds for $(X_1' = 1, X_2' = 1)$ is $\frac{0.4266}{1-0.4266} = 0.7440$. Thus, the assumption that the LR form is preserved when surrogate factors, rather than the true causal factors are observed (that is, that the correlation structure between the causal and surrogate factors can be accounted for while preserving the baseline LR model form) is contradicted in this example and, thus, as a general rule.

Appendix C: Theorems and Proofs of AIM

C.1 Stochastic data generation

The stochastic data generation mechanism for the disease status C given an observed factor vector $\underline{X} = \underline{x}$, is as follows: 1)Independently randomly generate C_i given x_i, $i = 1, \ldots, N$, according to the probability model defined in the method section of main body, and randomly generate C_0 according to the pmf $\{1 - \phi_0, \phi_0\}$. 2)Assign $C = 0$ if $(C_0 = 0) \cap (C_1 = 0) \cap (C_2 = 0) \cdots (C_N = 0)$. Otherwise, assign $C = 1$.

C.2 AIM model learning and hypothesis testing

In this section, we show that AIM's maximum likelihood (ML) objective is concave in the parameters $\underline{\beta}$, with the constrained ML learning problem (concave objective, linear constraints) thus a convex optimization problem[8], amenable to finding the global maximum. We propose a hybrid Newton-Barrier function algorithm for its solution. Statistical interaction detection for AIM, like LR, is based on a log-likelihood ratio statistic which, under the null, is asymptotically chi-squared[9].

Theorem 1: AIM's log-likelihood function is concave in its model parameters.

Proof: The Hessian matrix of second order partial derivatives $H = [\partial^2 \log L / \partial\beta_i \partial\beta_j] = -\sum_{i=1}^{M} I_i w_i \underline{y}_i \underline{y}_i^T$, where $\underline{y}_i = [1\ \underline{x}_i]$, I_i is an indicator with value 1 if subject i is a case and zero otherwise, and $w_i = P_{\text{AIM}}(C = 1|\underline{x}_i)/(1 - P_{\text{AIM}}(C = 1|\underline{x}_i))^2 > 0$. A non-negatively weighted outer product is non-negative definite, and a sum of non-negative definite matrices is also non-negative. Thus, the Hessian matrix (with a negative sign out front) is non-positive definite. Further, assuming the matrix is full rank, it is negative definite. Thus, the log-likelihood function is concave in its parameters.

C.3 Constrained maximum likelihood algorithm for estimating the AIM model

As shown in last section, AIM's log-likelihood objective function is concave in the parameters $\underline{\beta}$. Since the linear constraints on these parameters form a convex feasible region in the parameter space, constrained MLE of AIM's model parameters amounts to a convex optimization[8], for which globally optimal parameter estimates (or those approximating the global optimum to any required level of precision) can be efficiently found. In the sequel, we describe the two-step algorithm[8], based

on use of Newton-Raphson as initialization and a log-barrier interior point algorithm[8] to refine the solution, which we used in producing AIM MLE parameter estimates.

Assume M subjects, with $I_i = 1$ indicating the i-th subject is a case and $I_i = 0$ indicating a control. Define the augmented factor vector $\underline{y} = [1 \;\; \underline{x}^T]$. Thus, given the parameter vector $\underline{\beta}$, the class posterior log-likelihood over all M subjects (assuming subject independence) is:

$$L(\underline{\beta}) = \sum_{i=1}^{M} \left((1 - I_i)\underline{\beta}^T \underline{y}_i + I_i \log(1 - e^{\underline{\beta}^T \underline{y}_i})\right). \tag{4}$$

Maximum likelihood estimation for $\underline{\beta}$ is posed as:

$$\arg\max_{\underline{\beta}} L(\underline{\beta}) \text{ subject to } \underline{\beta}^T \underline{y}_i \leq 0, i = 1, \ldots, M. \tag{5}$$

Note that even if $M > 2^N$, where N is the number of factors in consideration, the number of distinct constraints is upper-bounded by the number of unique binary factor vectors, 2^N.

C.3.1 Optimization strategy

An important empirical observation is that, frequently, the solution to the unconstrained problem (obtained by ignoring the constraints in (5)) in fact satisfies all the constraints. We thus propose a two-stage optimization exploiting this to achieve computational efficiency in practice. In the first stage, Newton-Raphson is used to solve the unconstrained MLE problem, albeit with a check on constraint satisfaction after each iteration. If any constraint is violated, we terminate this stage and go to the second stage; otherwise, Newton-Raphson iterations are applied until the specified convergence target is met. In the provisional second stage, we apply the log-barrier method[8] to solve the constrained problem. The basic idea is to define a penalized log-likelihood function that leaves the log-likelihood unmodified when there are no constraint violations but severely penalizes any violations. The penalty function thus acts as a "barrier", ensuring the parameter vector always remains feasible while iteratively maximizing the penalized objective starting from the interior of the feasible region. Since the penalty function described above is not in general differentiable, we instead construct a differentiable surrogate penalty, indexed by a parameter t, that approaches the desired penalty in the limit as $t \to \infty$. The modified penalized log-likelihood (based on the surrogate penalty) is maximized for an increasing sequence of parameter values $t^{(0)}, t^{(1)}, t^{(2)}, \ldots$ via a continuation method, *i.e.*, with the solution for the objective indexed by $t^{(i)}$ used as initialization for $t^{(i+1)}$. From the duality theory of convex optimization[8], one can strictly bound the log-likelihood deficiency of the current solution (with respect to the global maximum log-likelihood),

which inversely depends on t. Thus, one can achieve any desired precision to the global maximum by optimizing for t sufficiently large. This log-barrier approach is described in detail in reference 8. Below, we summarize the main algorithm steps.

C.3.2 First stage: Newton-Raphson to solve the unconstrained problem

Given the current estimate $\underline{\beta}^{(k)}$, the next estimate $\underline{\beta}^{(k+1)}$ is produced by:

$$\underline{\beta}^{(k+1)} = \underline{\beta}^{(k)} - H(\underline{\beta}^{(k)})\nabla_{\underline{\beta}} L(\underline{\beta}^{(k)}),$$

where $H(\cdot)$ is the Hessian matrix:

$$H(\underline{\beta}) = \sum_{i=1}^{M} I_i P_{\mathrm{AIM}}(C=1|\underline{x}_i;\underline{\beta})/(1-P_{\mathrm{AIM}}(C=1|\underline{x}_i;\underline{\beta}))^2 \underline{y}_i \underline{y}_i^T \tag{6}$$

and

$$\nabla_{\underline{\beta}} L(\underline{\beta}) = \sum_{i=1}^{M} (1-I_i)\underline{y}_i + I_i P_{\mathrm{AIM}}(C=1|\underline{x}_i;\underline{\beta})/(1-P_{\mathrm{AIM}}(C=1|\underline{x}_i;\underline{\beta}))\underline{y}_i \underline{y}_i^T. \tag{7}$$

The Newton method has a quadratic convergence rate. It usually converges in less than 10 iterations in our application.

C.3.3 Second stage: Barrier method to solve the constrained problem

Letting $I_+(x) = 0$ if $x > 0$ and ∞ otherwise, the preceding constrained optimization problem is equivalent to: $\max_{\underline{\beta}} L(\underline{\beta}) + \sum_{i=1}^{M} I_+(-\underline{\beta}^T \underline{y}_i)$. Note that the optimum must occur for $\underline{\beta}$ satisfying $\underline{\beta}^T \underline{y}_i < 0, i = 1, \ldots, M$ to avoid the infinite penalty. Moreover, in this (feasible) case, the penalized log-likelihood reduces to the pure log-likelihood. Since $I^+(x)$ is not differentiable, we substitute the following penalty function that approaches $I^+(x)$ in the limit of large t: $\phi_t(x) = \frac{1}{t}\log(x)$ for $x > 0$ and ∞ otherwise. For given t, we thus solve: $\max_{\underline{\beta}} L(\underline{\beta}) + \sum_{i=1}^{M} \phi_t(-\beta^T \underline{y}_i)$. Note that this objective function is also concave. Thus, for each t, we can apply the Newton method for its maximization. As $t \to \infty$, this modified problem approaches the original problem.

C.3.4 Schedule for t and choice of $t^{(0)}$

The control parameter is updated using an exponential schedule: $t^{(k+1)} = \lambda t^{(k)}$, $\lambda > 1$. As discussed in reference 8, a reasonable choice for $t^{(0)}$ is such that $M/t^{(0)}$ is approximately $\lambda(L^* - L(\underline{\beta}^{(0)})$, where L^* is the true maximum. Since L^* and $L(\underline{\beta}^{(0)})$ are both unknown, we approximate their difference

by the log-likelihood difference between the last two Newton iterations in stage 1 (immediately prior to detecting constraint infeasibility).

While large λ reduces the number of optimizations performed in reaching a target value t_{final}, a "too large" difference $(t^{(k+1)} - t^{(k)})$ may mean that the solution at step k gives poor initialization at step $k+1$, translating to slow convergence of the Newton algorithm. There is thus a tradeoff in the choice of λ. Experiments suggest that values in the range $[3, 100]$ are reasonable choices. In our experiments we set $\lambda = 20$.

C.3.5 Parameter choices

For the inner loop (Newton minimization), we stop if the increase in log-likelihood is less than 10^{-6}. For the outer loop (over t), we stop if $t^{(k)} > 10^6 M$. From the dual optimization theory[8], this ensures a log-likelihood deficiency of less than 10^{-6}.

C.4 Link to well-established biological theories of disease

In addition to its asymmetry, AIM is supported by several well-accepted biological models, including the heterogeneity theory[10] and the two-hits theory of cancer[11]. The former states that any one of many different mutations in any one of many different genes leads to related phenotypes. Take hearing loss[10] as an example. Here, the responsible genes encode proteins involved in a wide variety of processes in the inner ear, including development and maintenance of cytoskeletal structures, myosin motors, gap junction transport and signaling, ion channels, and transcriptional regulators. Consistent with our assumption that an overall healthy occurs only if every active factor does not cause its local status to be 'diseased' in our model, if any of these processes fails, the person will lose hearing. It is also reasonable to have the assumption that factors independently exert effects, due to separation of the functional modules. The two-hits theory of cancer makes this assumption even more compelling. When one individual possesses some disease-risk factors (often germline mutations), this is called the first hit. Disease will not develop until the second hit – random somatic mutation – occurs. Hence, each disease-risk factor exerts its effect through random somatic mutation. Moreover, in general, random somatic mutations for different genes are expected to be independent.

While we have argued that AIM is more biologically plausible than LR, compelling support also comes from the *invariance* of this model, unlike LR, in the presence of unmeasured, surrogate factor, and disease heterogeneity (subtype) confounding effects.

C.5 Model consistency in the presence of both unmeasured and surrogate risk factors

The pervasiveness of missing and surrogate factors (as previously discussed) raises fundamental questions for models of joint baseline effects: 1) Is the model consistent (i.e. is the model's form preserved) when there are missing and surrogate risk factors? 2) If the model form is not preserved, what are the performance implications? We will resolve 2) empirically through our experiments. To resolve 1), we have the following theorems, proved in Appendices C.5-C.7.

Theorem 2: Assuming statistically independent factors, AIM is a consistent model when there are unmeasured (missing) causal factors, while LR is not. Moreover, under AIM, the parameter values themselves ($\{\beta_i\}$) for the observed factors do not change, in the presence of missing factors.

Proof: Suppose that the N factors $X_i, i = 1, \ldots, N$, each with discrete range denoted $\mathcal{R}(X_i)$, are statistically independent and that, given all factors observed, the disease status is generated according to the AIM posterior $P_{\text{AIM}}(C = 1|\underline{x}) = 1 - P(C = 0|\underline{x}) = 1 - e^{\beta_0 + \sum_{i=1}^{N} \beta_i x_i}$, where β_0 will be referred to as the background parameter. We prove that the posterior on disease status remains of this form when only a subset of the factors are observed. Let $\mathcal{S} \subset \{1, 2, \ldots, N\}$ be the indexes of the observed factors. We thus show that, for any $\mathcal{S}$, $\log(1 - P(C = 1|\underline{x})) = e^{\beta'_0 + \sum_{i \in \mathcal{S}} \beta'_i x_i}$, where, *moreover*, $\beta'_i = \beta_i, i \in \mathcal{S}$.

Let $\mathcal{S}_j$ be any subset of cardinality j. The proof is by induction on j. First, note that $\mathcal{S}_N = \{1, 2, \ldots, N\}$ and, since by assumption the posterior is the AIM model when all factors are observed, for $\mathcal{S}_N$ the posterior is indeed of the AIM form, with $\beta'_i = \beta_i, i = 1, \ldots, N$ and $\beta'_0 = \beta_0$. Thus, the results holds at $j = N$. Next, assume that the result holds for any subset of size j, $\mathcal{S}_j = \{i_1, i_2, \ldots, i_j\}$. That is, the posterior on disease status, given observation of the factors in the subset $\mathcal{S}_j$, is of the AIM form, with unperturbed parameter values $\beta'_i = \beta_i, i \in \mathcal{S}_j$. Let us denote the background parameter value in this posterior by $\tilde{\beta}_0$. We must show consequentially the result also holds for the subsets $\mathcal{S}_{j-1}$. Note that if we remove one factor from any subset $\mathcal{S}_j$, we obtain a subset $\mathcal{S}_{j-1}$. We can express the posterior for this subset by:

$$\begin{aligned} & P[C = 1|x_{i_1}, x_{i_2}, \ldots, x_{i_{j-1}}] & (8) \\ = \; & \textstyle\sum_{x_{i_j} \in \mathcal{R}(X_{i_j})} P[C = 1, x_{i_j}|x_{i_1}, x_{i_2}, \ldots, x_{i_{j-1}}] \end{aligned}$$

$$
\begin{aligned}
&= \sum\nolimits_{x_{i_j} \in \mathcal{R}(X_{i_j})} P[C=1|x_{i_1}, x_{i_2}, \ldots, x_{i_{j-1}}, x_{i_j}] P[X_{i_j} = x_{i_j} | x_{i_1}, x_{i_2}, \ldots, x_{i_{j-1}}] \\
&= \sum\nolimits_{x_{i_j} \in \mathcal{R}(X_{i_j})} P[C=1|x_{i_1}, x_{i_2}, \ldots, x_{i_{j-1}}, x_{i_j}] P[X_{i_j} = x_{i_j}] \\
&= \sum\nolimits_{x_{i_j} \in \mathcal{R}(X_{i_j})} (1 - e^{\tilde{\beta}_0 + \sum_{l=1}^{j} \beta_{i_l} x_{i_l}}) P[X_{i_j} = x_{i_j}] \\
&= 1 - \Big(\sum\nolimits_{x_{i_j} \in \mathcal{R}(X_{i_j})} e^{\beta_{i_j} x_{i_j}} P[X_{i_j} = x_{i_j}] \Big) e^{\tilde{\beta}_0 + \sum_{l=1}^{j-1} \beta_{i_l} x_{i_l}} \\
&= 1 - e^{c + \tilde{\beta}_0 + \sum_{l=1}^{j-1} \beta_{i_l} x_{i_l}},
\end{aligned}
$$

where $c = \log(\sum_{x_{i_j} \in \mathcal{R}(X_{i_j})} e^{\beta_{i_j} x_{i_j}} P[X_{i_j} = x_{i_j}])$. So, the posterior's form is preserved, with $\beta'_{i_l} = \beta_{i_l}, i_l \in \mathcal{S}_{j-1}$ and where we identify the new background parameter, with no dependence on any of the 'observed' factors, as $\beta'_0 = \tilde{\beta}_0 + \log(\sum_{x_{i_j} \in \mathcal{R}(X_{i_j})} e^{\beta_{i_j} x_{i_j}} P[X_{i_j} = x_{i_j}])$.
Q.E.D.

Theorem 3: Assume that some causal factors are not measured, but surrogate factors, correlated with these true factors are instead measured. Assume the following statistical dependency structure: a causal factor X_i is conditionally independent of all other factors (either the true factors or their surrogates), given the causal factor's surrogate, X_i^0. Also assume that the disease status is conditionally independent of a surrogate factor, given the true factor. Then, AIM is consistent under the surrogate factors scenario, while LR is not. Moreover, under AIM, the parameter values themselves ($\{\beta_i\}$) for the observed true factors do not change, in the presence of surrogate factors.

Proof: Suppose there are N true disease factors X_i, each with discrete range space $\mathcal{R}(X_i), i = 1, \ldots, N$, and with one special value of the range space, denoted v_i, corresponding to the disease factor not being *active*[1]. Suppose that, when all N factors are observed, the disease status posterior has the AIM form:

$$\log(1 - P(C=1|x_1, x_2, \ldots, x_N)) = \beta_0 + \sum\nolimits_{i=1}^{N} \beta_i(x_i), \tag{9}$$

where if $x_i = v_i$, $\beta_i(x_i) = 0, \forall i$. Now suppose that there is a subset of factors which are *not* observed. However, rather than being missing, *surrogate* factors, correlated with these true factors, are observed. Let X'_i denote the observed surrogate factor correlated with true factor X_i. Assume

[1]Here we are allowing each factor to have a non-binary range space. In the case of binary factors, consistent with the derivation of the AIM model in the main paper, the value indicating a factor's inactivity is $v_i = 0$. More generally, for non-binary factors, we are supposing there is a value v_i indicative of a factor's inactivity.

each true factor X_i is conditionally independent of all other factors (true or surrogate) given its surrogate X'_i. Further, assume that the disease status is conditionally independent of a surrogate factor given its true factor. Under these assumptions, we prove that the posterior probability on disease status, given all observed factors (both true and surrogate factors) *remains* of the AIM form and, moreover, is such that, for a true observed factor $X_i = x_i$, its parameter value is $\beta_i(x_i) \forall x_i \in \mathcal{R}(X_i)$, that is the parameter value is *unaltered* by the presence of surrogate factors.

Let $\mathcal{S}_j = \{i_1, i_2, \ldots, i_j\}$ be any subset of surrogate factors, of cardinality j, with companion set $\bar{\mathcal{S}}_j = \{l_1, l_2, \ldots, l_{N-j}\}$. The proof is by induction on j. First, note that $S_0 = \{\}$, that is, all true factors observed. Since by assumption the posterior in this case has the AIM form with parameter values $\beta_i, i = 1, \ldots, N$, the result holds for $j = 0$. Next, assume that the result holds for some $j > 0$, *i.e.*, for the subsets $\mathcal{S}_j$. That is, given j observed surrogate factors and $N - j$ observed true factors, the posterior form is the AIM form, where, further, for each true factor value $X_i = x_i$, its parameter value is $\beta_i(x_i)$, *i.e.*, the same value as when no observed factors are surrogates. Denoting the set of parameter values for a surrogate factor X'_i by $\beta'_i(\omega), \omega \in \mathcal{R}(X'_i)$, the posterior form for any surrogate factor subset $\mathcal{S}_j$ is thus:

$$\begin{aligned} \log(1 - P(C = 1|x'_{i_1}, x'_{i_2}, \ldots, x'_{i_j}, x_{l_1}, x_{l_2}, \ldots, x_{l_{N-j}})) = \\ \tilde{\beta}_0 + \sum_{m=1}^{j} \beta'_{i_m}(x'_{i_m}) + \sum_{n=1}^{N-j} \beta_{l_n}(x_{l_n}), \end{aligned} \tag{10}$$

where $\tilde{\beta}_0$ is the value of the background parameter (which will not in general equal β_0).

We must show this result consequentially holds for the subsets $\mathcal{S}_{j+1}$. The posterior for a subset $\mathcal{S}_{j+1}$ is:

$$\begin{aligned} & P[C = 0|x'_{i_1}, x'_{i_2}, \ldots, x'_{i_{j+1}}, x_{l_1}, x_{l_2}, \ldots, x_{l_{N-j-1}}] \\ = & \sum_{x_{i_{j+1}} \in \mathcal{R}(X_{i_{j+1}})} P[C = 0, x_{i_{j+1}}|x'_{i_1}, x'_{i_2}, \ldots, x'_{i_{j+1}}, x_{l_1}, x_{l_2}, \ldots, x_{l_{N-j-1}}] \\ = & \sum_{x_{i_{j+1}} \in \mathcal{R}(X_{i_{j+1}})} P[C = 0|x'_{i_1}, x'_{i_2}, \ldots, x'_{i_{j+1}}, x_{l_1}, x_{l_2}, \ldots, x_{l_{N-j-1}}, x_{i_{j+1}}] \times \\ & \qquad P[X_{i_{j+1}} = x_{i_{j+1}}|x'_{i_1}, x'_{i_2}, \ldots, x'_{i_{j+1}}, x_{l_1}, x_{l_2}, \ldots, x_{l_{N-j-1}}] \\ = & \sum_{x_{i_{j+1}} \in \mathcal{R}(X_{i_{j+1}})} P[C = 0|x'_{i_1}, x'_{i_2}, \ldots, x'_{i_j}, x_{i_{j+1}}, x_{l_1}, x_{l_2}, \ldots, x_{l_{N-j-1}}] P[X_{i_{j+1}} = x_{i_{j+1}}|x'_{i_{j+1}}] \\ = & \sum_{x_{i_{j+1}} \in \mathcal{R}(X_{i_{j+1}})} e^{\tilde{\beta}_0 + \sum_{n=1}^{N-j-1} \beta_{l_n}(x_{l_n}) + \sum_{m=1}^{j} \beta'_{i_m}(x'_{i_m})} e^{\beta_{i_{j+1}}(x_{i_{j+1}})} P[X_{i_{j+1}} = x_{i_{j+1}}|x'_{i_{j+1}}] \\ = & e^{\tilde{\beta}_0 + \sum_{n=1}^{N-j-1} \beta_{l_n}(x_{l_n}) + \sum_{m=1}^{j} \beta'_{i_m}(x'_{i_m})} \left(\sum_{x_{i_{j+1}} \in \mathcal{R}(X_{i_{j+1}})} e^{\beta_{i_{j+1}}(x_{i_{j+1}})} P[X_{i_{j+1}} = x_{i_{j+1}}|x'_{i_{j+1}}] \right) \\ = & e^{\tilde{\beta}_0 + \sum_{n=1}^{N-j-1} \beta_{l_n}(x_{l_n}) + \sum_{m=1}^{j} \beta'_{i_m}(x'_{i_m}) + \beta'_{i_{j+1}}(x'_{i_{j+1}})}. \end{aligned} \tag{11}$$

Here, the third resultant is obtained using the fact that disease status is conditionally independent of $X'_{i_{j+1}}$ given $X_{i_{j+1}}$ and the fact that $X_{i_{j+1}}$ is conditionally independent of all other factors given $X'_{i_{j+1}}$. The fourth resultant is obtained because, by assumption, the result holds for the subsets $\mathcal{S}_j$. Finally, in the final result, we have made the identification that $\beta'_{i_{j+1}}(x'_{i_{j+1}}) = \sum_{x_{i_{j+1}} \in \mathcal{R}(X_{i_{j+1}})} e^{\beta_{i_{j+1}}(x_{i_{j+1}})} P[x_{i_{j+1}}|x'_{i_{j+1}}]$, *i.e.*, a quantity that is a function only of $x'_{i_{j+1}}$. Thus, the posterior's form is preserved for subsets of size $j+1$.

Q.E.D.

Comments:

1) For LR, the results are proved by counterexample (as already shown in the main body for the unmeasured factors scenario). 2) In the unmeasured case, the new model form is obtained by marginalizing (integrating out) unmeasured factors. Marginalization of AIM leads to the same mathematical model form, while this is not true for LR. To understand why the AIM form is preserved under unmeasured factors, note that $P_{\text{AIM}}(C = 0|\underline{x}) = e^{\beta_0} \prod_{i=1}^{N} e^{\beta_i x_i}$. Thus, when a factor is not measured, it is essentially omitted from the product – this effects marginalization, and preserves AIM's log-additive form on the remaining factors. The practical implication of these theorems, demonstrated in Appendix D, is that LR has inflated type 1 error under these scenarios, while AIM does not. Moreover, AIM has greater detection power than LR under these scenarios; 3) The rigorous proofs of Theorems 2 and 3 require the assumption of independence between factors. This assumption may not hold for some applications. We use simulations to investigate the implications of the violation of this assumption. As shown in Appendix D, we do not observe any inflation in type I error rate when there are missing factors, suggesting Theorem 2 remains practically valid. On the other hand, we do see that Theorem 3 cannot be true when the independence assumption is violated. However, the effect is not detectable when the correlation between factors is moderate, and the effect is still small when the correlation is very strong.

C.6 Model consistency under disease heterogeneity

AIM is *also* a consistent model, and LR an inconsistent one, with respect to yet another confounding source – disease *heterogeneity*. Specifically, suppose that there are several disease subtypes $D_i \in \{0, 1\}, i = 1, \ldots, K$, where $D_i = 1$ means the i-th subtype is present in an individual. Likewise, the heterogeneous disease is present, i.e. $C = 1$, if and only if at least one disease subtype is present, i.e. if and only if $(D_1 = 1) \bigcup (D_2 = 1) \cdots \bigcup (D_K = 1)$. If the different subtypes are known *and* if the

cases in the population were ground-truth labeled by subtype, one could estimate a separate case-control posterior model quantifying the baseline risk for each disease subtype $P(D_i|\underline{x})$. However, in practice, this is unrealistic – a complex disease may decompose as subtypes, but these will typically be *latent*, with explicit knowledge only of whether the heterogeneous disease is present, not which subtype. Regardless of whether subtypes are explicitly known or not, there is a posterior for each disease subtype $P(D_i|\underline{x}), i = 1, \ldots, K$. Moreover, a model for the complex disease status is the posterior $P(C = 1|\underline{x}) = P((D_1 = 1)\bigcup(D_2 = 1)\cdots\bigcup(D_K = 1)|\underline{x})$. If the individual subtype models are AIM models, and if disease subtypes are conditionally independent given the observed factors, then one can show that the complex disease model is *also* an AIM model, i.e. the AIM parametric form is invariant to disease heterogeneity. On the other hand, this is again not true for LR. Specifically, we have:

Theorem 4: Suppose that a complex disease contains multiple subtypes, which are assumed to be conditionally independent given the observed factors. Then, the AIM model form is invariant to disease heterogeneity, i.e. $P((D_1 = 1)\bigcup(D_2 = 1)\cdots\bigcup(D_K = 1)|\underline{x}) = P_{\text{AIM}}(C = 1|\underline{x})$**, where, in particular, the weight** β_i **on an individual factor** X_i **in the heterogeneous AIM model is** *additive* **over the weights on this factor for each of the disease subtype models. On the other hand, the LR form is not invariant to disease heterogeneity.**

Proof: Here we will only prove that AIM *is* a consistent model under the scenario of a heterogeneous disease with multiple subtypes. While not shown here, the inconsistency of LR as a model for a heterogeneous disease can be proven by counterexample, just as we have done for the missing and surrogate factor confounding scenarios.

Suppose that there are K disease subtypes, each with a disease subtype status posterior of the AIM form:

$$\log(1 - P(D_k = 1|x_1, x_2, \ldots, x_N)) = \beta_{k0} + \sum_{i=1}^{N} \beta_{ki}x_i, k = 1, \ldots, K. \tag{12}$$

Further, assume that these subtypes are conditionally independent given the observed factors $X_i, i = 1, \ldots, N$. Under these assumptions, we will prove that the heterogeneous disease status $C = \bigcup_{k=1}^{K} D_k$ has a posterior that is also of the AIM form, *i.e.*,

$$\log(1 - P(C = 1|x_1, x_2, \ldots, x_N)) = \beta_0 + \sum_{i=1}^{N} \beta_i x_i. \tag{13}$$

Furthermore, $\beta_i = \sum_{k=1}^{K} \beta_{ki}, i = 1, \ldots, N$ and $\beta_0 = \sum_{k=1}^{K} \beta_{k0}$. That is, the strengths of each of the factors for the heterogeneous disease is additive over the strengths for each of the subtypes. We note an important implication of this result: to do inference on the heterogeneous disease using the AIM model, one need not have *any* prior knowledge of how many (and whether in fact) multiple disease subtypes exist for the given disease domain. On the other hand, since the LR form (if assumed to be valid for the subtypes) is not preserved for the heterogeneous disease, such statement will not hold for LR.

The proof of the theorem is as follows. We have $(C = 0) \Leftrightarrow \bigcap_{k=1}^{K}(D_k = 0)$. Therefore, $P(C = 0|x_1, x_2, \ldots, x_N) = P(D_1 = 0, D_2 = 0, \ldots, D_K = 0|x_1, x_2, \ldots, x_N)$. But since the disease subtypes are conditionally independent given the factors, $P(D_1 = 0, D_2 = 0, \ldots, D_K = 0|x_1, x_2, \ldots, x_N) = \prod_{k=1}^{K} P(D_k = 0|x_1, \ldots, x_N)$. Thus,

$$\begin{aligned} \log(P(C = 0|x_1, x_2, \ldots, x_N) &= \sum_{k=1}^{K} \log(P(D_k = 0|x_1, \ldots, x_N) \\ &= \sum_{k=1}^{K} (\beta_{k0} + \sum_{i=1}^{N} \beta_{ki} x_i) \\ &= (\sum_{k=1}^{K} \beta_{k0}) + \sum_{i=1}^{N} x_i (\sum_{k=1}^{K} \beta_{ki}) \\ &= \beta_0 + \sum_{i=1}^{N} x_i \beta_i, \end{aligned} \tag{14}$$

where $\beta_0 = \sum_{k=1}^{K} \beta_{k0}$ and $\beta_i = \sum_{k=1}^{K} \beta_{ki}$.
Q.E.D.

An important implication of this theorem is the following: to do inference on the heterogeneous disease using the AIM model, one need not have *any* prior knowledge of how many (and whether in fact) multiple disease subtypes exist for the given disease domain. The AIM modeling approach is naturally accommodating of however many disease subtypes that may be present (through the additive weight mechanism).

C.7 Theoretical characterization of interaction detection power for AIM and LR

Generally speaking, for a two-sided hypothesis testing problem it is difficult to draw a uniform conclusion on the power comparison between two competing models. In fact, in general a "no free lunch theorem" should apply, with no model/method uniformly dominating another. Thus, it is very useful (but often extremely difficult) to identify the conditions under which one model is theoretically guaranteed to outperform another. Such results can inform when it is most suitable in practice to apply one model, rather than another. The most important contribution of this

paper is that we have identified conditions under which AIM is guaranteed to perform better than LR. Strongly supporting the usefulness of AIM, these conditions correspond to the *most common* scenarios encountered in real applications. Consider two types of interactions: (1) synergistic and (2) antagonistic. A synergistic interaction means that the true effect associated with the joint occurrence of two risk factors is greater than a baseline model's (without interaction) joint effect[12]. On the other hand, if the true joint effect is smaller than a baseline model's (without interaction) joint effect, we call it "antagonistic". Most interactions found in practice are synergistic[12].Theorem 5 below, based on a precise and meaningful definition of synergistic interactions, shows that AIM has better power to detect synergistic interactions than LR. Somewhat surprisingly, this statement holds even if the ground-truth disease status generating model is an LR model with interaction terms. Thus, this result defies, at least with respect to detection power, the credo that it is optimal to match the chosen model to the data-generating mechanism. A corollary can also be derived stating that LR is guaranteed better power than AIM for antagonistic interactions. However, even for antagonistic interactions we argue against the use of LR because of its degraded performance when there are missing and surrogate factors and/or disease subtypes.

Theorem 5: Given two binary variables X_1 and X_2, let $p_{00} \equiv P(C = 1|X_1 = 0, X_2 = 0)$ be the true posterior probability and similarly define p_{01}, p_{10}, and p_{11}. Assume $p_{01} \geq p_{00}$ and $p_{10} \geq p_{00}$. Denote p'_{11} the predicted value from the LR model whose three parameters are determined *solely* by the true posterior probabilitiesp_{00}, p_{10}, and p_{01}. We define a synergistic interaction as one satisfying $p_{11} \geq p'_{11}$. Under the above assumptions, we then have the following result: for synergistic interactions, the AIM model learned on a given population gives a greater difference between its interaction model and baseline model log likelihoods on that population than that for the LR model learned on the same population. Hence, AIM generates a strictly smaller p-value than LR and hence has better power to detect interaction effects than LR. Likewise, if $p_{11} < p'_{11}$, *i.e.* an antagonistic interaction, then LR generates a smaller p-value than AIM.

Proof: Suppose there are $N = 2$ binary factors. Let $p_{lm} \equiv P(C = 1|X_1 = l, X_2 = m), l \in \{0, 1\}, m \in \{0, 1\}$ be the true posterior probability of disease, for each of the four possible factor combinations. We will prove that, if $p_{10} \geq p_{00}$ and $p_{01} \geq p_{00}$, then, for synergistic interactions (defined by $p_{11} \geq p'_{11}$ as given in Theorem 5), AIM has a greater difference between its interaction

model and baseline model log-likelihoods than that for LR. Accordingly, since for both models the log-likelihood difference (distributed as chi-squared with the same number of degrees of freedom) is used to assess statistical significance of an interaction, the AIM model will produce strictly smaller p-values than the LR model for synergistic interactions.

The proof exploits the fact that there are several ways one can determine the parameters of a logistic regression model. One way is of course to estimate the model parameters to maximize the population data log-likelihood. However, an alternative way to estimate LR parameter values is to determine them so as to be *strictly consistent with* given posterior probabilities q_{00}, q_{10}, and q_{01}. In particular, we note that, based on the LR model form $\log(P(C=1|X_1,X_2)/(1-P(C=1|X_1,X_2)) = \alpha_0+\alpha_1X_1+\alpha_2X_2$. Thus, for the LR model, strict consistency with q_{00}, q_{10}, and q_{01} means that: $\frac{q_{00}}{1-q_{00}} = e^{\alpha_0}$, $\frac{q_{10}}{1-q_{10}} = e^{\alpha_0+\alpha_1}$, and $\frac{q_{01}}{1-q_{01}} = e^{\alpha_0+\alpha_2}$. Thus, $\alpha_0 = \log(\frac{q_{00}}{1-q_{00}})$, $\alpha_1 = \log(\frac{q_{10}}{1-q_{10}}) - \log(\frac{q_{00}}{1-q_{00}})$, and $\alpha_2 = \log(\frac{q_{01}}{1-q_{01}}) - \log(\frac{q_{00}}{1-q_{00}})$.

Our proof of Theorem 5 is based on a consideration of three different LR models: i) the maximum likelihood LR model; ii) the surrogate LR model with parameters determined by the *true* posteriors, *i.e.*, $q_{00} = p_{00}$, $q_{10} = p_{10}$, and $q_{01} = p_{01}$; iii) the surrogate LR model with parameters determined by the maximum likelihood AIM model's posteriors (denoted $p_{lm}^{(A)}$), *i.e.*, $q_{00} = p_{00}^{(A)}$, $q_{10} = p_{10}^{(A)}$, and $q_{01} = p_{01}^{(A)}$.

The proof structure is as follows. We first consider the surrogate LR model (LR') whose parameter values are determined by the (maximum likelihood) AIM model. Lemma 5.1 below establishes some key results concerning this surrogate LR model and the maximum likelihood AIM model. The next step is to establish a result (Lemma 5.2) that essentially says that a new model, formed by mixing a given model's probabilities with the true probabilities, necessarily has greater data log-likelihood than the original, given model. Finally, we exploit these Lemmas, along with the synergistic interaction assumption, to establish our (desired) detection power results. After stating Lemma 5.1 and Lemma 5.2, we proceed with the proof of Theorem 5.

C.7.1 Lemma 5.1:

Let $p_{00}^{(A)}, p_{01}^{(A)}, p_{10}^{(A)}, p_{11}^{(A)}$ denote the posterior disease probabilities, under the four factor combinations, for the baseline AIM model $\log(1-P(C=1|\underline{X})) = \beta'_0+\beta'_1X_1+\beta'_2X_2$, where the parameter values $\beta'_0, \beta'_1, \beta'_2$ maximize the model's data log-likelihood on the given population. We have the following results: 1) For the LR model (denoted LR') whose parameters are determined based on $p_{00}^{(A)}, p_{01}^{(A)}, p_{10}^{(A)}$, we have $p_{00}^{(LR')} = p_{00}^{(A)}, p_{01}^{(LR')} = p_{01}^{(A)}, p_{10}^{(LR')} = p_{10}^{(A)}$; 2) *Ordering Property:* Assuming

$p_{10} \geq p_{00}$, $p_{01} \geq p_{00}$, $p_{11} \geq p_{10}$, and $p_{11} \geq p_{01}$, it follows that $p_{10}^{(A)} \geq p_{00}^{(A)}$ and $p_{01}^{(A)} \geq p_{00}^{(A)}$, *i.e.*, the MLE AIM model preserves the *ordering* of these posterior probabilities; 3) Under the same assumptions as 2), $p_{11}^{(LR')} \geq p_{11}^{(A)}$ with equality if and only if $p_{01}^{(A)} = p_{00}^{(A)}$ or $p_{10}^{(A)} = p_{00}^{(A)}$.

C.7.2 Lemma 5.2:

Consider an M-category phenotype ($M \geq 2$), taking on values $\{\omega_1, \ldots, \omega_M\}$, and a population of individuals of size $N = \sum_{m=1}^{M} N_m$, N_m the number of individuals possessing phenotype ω_m. Let $\mathcal{Q} = \{q_m, m = 1, \ldots, M\}$ be a probability mass function model for the phenotype, and let $\mathcal{P} = \{p_m \equiv \frac{N_m}{N}, m = 1, \ldots, M\}$, *i.e.*, it is the empirical pmf. The data log-likelihood for the population, under the model $\mathcal{Q}$, is: $L = \sum_{m=1}^{M} N_m \log(q_m) = N \sum_{m=1}^{M} p_m \log(q_m)$. Consider the new model $\mathcal{Q}' = \{\lambda p_m + (1-\lambda) q_m, m = 1, \ldots, M\}$, where $0 \leq \lambda \leq 1$, with log-likelihood $L' = N \sum_{m=1}^{M} p_m \log(q'_m)$. Then, $L' \geq L$ with equality iff $\mathcal{Q}' = \mathcal{Q}$.

C.7.3 Proof of Theorem 5 using Lemmas 5.1 and 5.2:

We now prove Theorem 5, making use of Lemmas 5.1 and 5.2. We will only provide the proof here for the synergistic interaction case, since the proof strategy is very similar for antagonistic interactions. Let $N_{lm}, l = 0,1,\ m = 0,1$ denote the number of subjects in the population with combination $(X_1 = l, X_2 = m)$. Then, the log-likelihood under the baseline AIM model is: $L^{(A)} = \sum_{l=0,1} \sum_{m=0,1} N_{lm} \Big(p_{lm} \log(p_{lm}^{(A)}) + (1-p_{lm}) \log(1-p_{lm}^{(A)})\Big)$. Likewise, we have $L^{(LR')} = \sum_{l=0,1} \sum_{m=0,1} N_{lm} \Big(p_{lm} \log(p_{lm}^{(LR')}) + (1-p_{lm}) \log(1-p_{lm}^{(LR')})\Big)$. Now, since, from Lemma 5.1, $p_{00}^{(LR')} = p_{00}^{(A)}, p_{01}^{(LR')} = p_{01}^{(A)}, p_{10}^{(LR')} = p_{10}^{(A)}$, we have that

$$L^{(LR')} - L^{(A)} = N_{11}\Big(p_{11} \log(p_{11}^{(LR')}) + (1-p_{11}) \log(1-p_{11}^{(LR')})\Big) - N_{11}\Big(p_{11} \log(p_{11}^{(A)}) + (1-p_{11}) \log(1-p_{11}^{(A)})\Big), \tag{15}$$

a difference between two log-likelihoods, restricted to the subpopulation with $(X_1 = 1, X_2 = 1)$. We next consider the sign of the difference $L^{(LR')} - L^{(A)}$ under the two possible cases: $p_{11} \geq p_{11}^{(LR')}$ and $p_{11} < p_{11}^{(LR')}$.

First, suppose $p_{11} \geq p_{11}^{(LR')}$. Let $\lambda = (p_{11} - p_{11}^{(LR')})/(p_{11} - p_{11}^{(A)})$. Note that $\lambda \geq 0$ because $p_{11} \geq p_{11}^{(LR')}$ and $p_{11} \geq p_{11}^{(A)}$. Also, $\lambda < 1$ because $p_{11}^{(LR')} > p_{11}^{(A)}$ and, thus, $p_{11} - p_{11}^{(LR')} < p_{11} - p_{11}^{(A)}$. Further, one can verify that $(p_{11}^{(LR')}, 1-p_{11}^{(LR')}) = \lambda(p_{11}^{(A)}, 1-p_{11}^{(A)}) + (1-\lambda)(p_{11}, 1-p_{11})$. Thus, by Lemma 5.2, the log-likelihood $N_{11}(p_{11} \log(p_{11}^{(LR')}) + (1-p_{11}) \log(1-p_{11}^{(LR')}))$ is greater than the log-likelihood $N_{11}(p_{11} \log(p_{11}^{(A)}) + (1-p_{11}) \log(1-p_{11}^{(A)}))$ and, thus, $L^{(LR')} > L^{(A)}$. Finally,

the maximum likelihood LR model has a log-likelihood $L^{(LR)}$ at least as large as $L^{(LR')}$, *i.e.*, $L^{(LR)} \geq L^{(LR')} > L^{(A)}$.

Next, suppose $p_{11} < p_{11}^{(LR')}$. Let us construct the vector $\underline{p}(t) = (p_{00}(t), p_{10}(t), p_{01}(t))$, where $p_{lm}(t) = p_{lm} + t(p_{lm}^{(A)} - p_{lm})$. Note that $\underline{p}(0) = (p_{00}, p_{10}, p_{01})$ and $\underline{p}(1) = (p_{00}^{(A)}, p_{10}^{(A)}, p_{01}^{(A)})$. As shown in Lemma 5.1, the parameters of the LR model can be determined by these three probabilities and, thus, by the triple $\underline{p}(t)$ (for any $0 \leq t \leq 1$). Let us denote the resulting LR posterior probability, given $(X_1 = 1, X_2 = 1)$, by $p_{11}^{(LR')}(t)$, a continuous function of t. Now, note that $p_{11}^{(LR')}(1) = p_{11}^{(LR')} > p_{11}$. Also, $p_{11}^{(LR')}(0) = p'_{11} < p_{11}$, since this is just our definition of a synergistic interaction. We thus have that $p_{11}^{(LR')}(0) < p_{11} < p_{11}^{(LR')}(1)$. Since $p_{11}^{(LR')}(t)$ is a continuous function, by the intermediate value theorem, there must be some value t_c, $0 < t_c < 1$, such that $p_{11}^{(LR')}(t_c) = p_{11}$. Let us consider the log-likelihood for this model, $L^{(LR')}(t_c)$, which can be written as $L^{(LR')}(t_c) = \sum_{l=0,1} \sum_{m=0,1} L_{lm}^{(LR')}(t_c)$, where $L_{lm}^{(LR')}(t_c) = N_{lm}(p_{lm} \log(p_{lm}^{(LR')}(t_c)) + (1-p_{lm}) \log(1-p_{lm}^{(LR')}(t_c)))$. Now, for $(l,m) = (0,0), (0,1)$, and$(1,0)$, we have that $(p_{lm}^{(LR')}(t_c), 1 - p_{lm}^{(LR')}(t_c)) = t_c(p_{lm}^{(A)}, 1 - p_{lm}^{(A)}) + (1-t_c)(p_{lm}, 1 - p_{lm})$. Recalling that $0 < t_c < 1$ and applying Lemma 5.2, we have that $L_{lm}^{(LR')}(t_c) > L_{lm}^{(A)}$, $(l,m) = (0,0), (1,0), (0,1)$, where $L_{lm}^{(A)}$ is the log-likelihood for the $(X_1 = l, X_2 = m)$ subpopulation under the AIM posterior model. Finally, since $p_{11}^{(LR')}(t_c) = p_{11}$, we have $(p_{11}^{(LR')}(t_c), 1 - p_{11}^{(LR')}(t_c)) = \lambda(p_{11}, 1 - p_{11}) + (1-\lambda)(p_{11}^{(A)}, 1 - p_{11}^{(A)})$, where $\lambda = 1$. Thus, again applying Lemma 5.2, we have: $L_{11}^{(LR')}(t_c) > L_{11}^{(A)}$.

Since all four combination-conditioned log-likelihoods for the LR model determined based on $\underline{p}(t_c)$ are greater than their counterpart log-likelihoods for the maximum likelihood AIM model, we have, for the composite log-likelihoods, that $L^{(LR')}(t_c) > L^{(A)}$. Moreover, the maximum likelihood LR model has log-likelihood $L^{(LR)} \geq L^{(LR')}(t_c)$. Thus, we again obtain $L^{(LR)} > L^{(A)}$.

In summary, under both possible scenarios ($p_{11} \geq p_{11}^{(LR')}$ and $p_{11} < p_{11}^{(LR')}$), $L^{(LR)} > L^{(A)}$. Finally, for hypothesis testing, we look at the difference between the log-likelihood under the alternative hypothesis (where an interaction term $\beta_3 X_1 X_2$ is included in the model), L_{Alt}, and the baseline model log-likelihood. Now, under the alternative hypothesis, for *both* AIM and LR, the models are *saturated*, *i.e.*, there are four combination values and four free parameters. Thus, the AIM and LR alternative hypothesis maximum likelihood models have the same log-likelihood value L_{alt}[4], which must be at least as large as the baseline model log-likelihoods. Thus, $L_{alt} \geq L^{(LR)}$, and since $L^{(LR)} > L^{(A)}$, we have $L_{alt} - L^{(LR)} < L_{alt} - L^{(A)}$, *i.e.*, AIM gives a greater log-likelihood difference.

Q.E.D.

C.7.4 Proof of Lemma 5.1:

First Result: Under the AIM model, $p_{00}^{(A)} = 1 - e^{\beta_0'}$, $p_{10}^{(A)} = 1 - e^{\beta_0'+\beta_1'}$, and $p_{01}^{(A)} = 1 - e^{\beta_0'+\beta_2'}$. Now, consider the LR model whose parameters are determined based on the AIM model's posterior probabilities $p_{00}^{(A)}, p_{10}^{(A)}, p_{01}^{(A)}$, rather than based on the true disease posteriors p_{00}, p_{10}, p_{01}. Accordingly, we let $\beta_0 = \log(\frac{p_{00}^{(A)}}{1-p_{00}^{(A)}})$, $\beta_1 = \log(\frac{p_{10}^{(A)}}{1-p_{10}^{(A)}}) - \log(\frac{p_{00}^{(A)}}{1-p_{00}^{(A)}})$, and $\beta_2 = \log(\frac{p_{01}^{(A)}}{1-p_{01}^{(A)}}) - \log(\frac{p_{00}^{(A)}}{1-p_{00}^{(A)}})$. Based on these parameter value assignments, the LR posterior probabilities are:

$$p_{00}^{(LR')} = e^{\beta_0}/(1+e^{\beta_0})|_{\beta_0=\log(\frac{p_{00}^{(A)}}{1-p_{00}^{(A)}})} = p_{00}^{(A)} \tag{16}$$

$$p_{10}^{(LR')} = e^{\beta_0+\beta_1}/(1+e^{\beta_0+\beta_1})|_{\beta_0=\log(\frac{p_{00}^{(A)}}{1-p_{00}^{(A)}}),\beta_1=\log(\frac{p_{10}^{(A)}}{1-p_{10}^{(A)}})-\log(\frac{p_{00}^{(A)}}{1-p_{00}^{(A)}})} = p_{10}^{(A)}.$$

Likewise, it is also found that $p_{01}^{(LR')} = p_{01}^{(A)}$.

Q.E.D.

Second Result:

The log-likelihood under an AIM model is:

$$L = \sum_{l=0,1} \sum_{m=0,1} p_{lm} \log(p_{lm}^{(A)}), \tag{17}$$

where $p_{00}^{(A)} = 1 - e^{\beta_0}$, $p_{10}^{(A)} = 1 - e^{\beta_0+\beta_1}$, $p_{01}^{(A)} = 1 - e^{\beta_0+\beta_2}$, and $p_{11}^{(A)} = 1 - e^{\beta_0+\beta_1+\beta_2}$. The maximum likelihood AIM model satisfies the necessary optimality conditions: $\partial L/\partial\beta_i = 0, i = 0, 1, 2$. Taking derivatives, we find that these conditions are:

$$\frac{\partial L}{\partial \beta_0} = \sum_{l=0,1} \sum_{m=0,1} N_{lm} \left(\frac{p_{lm}^{(A)} - p_{lm}}{p_{lm}^{(A)}} \right) = 0 \tag{18}$$

$$\frac{\partial L}{\partial \beta_1} = N_{10} \left(\frac{p_{10}^{(A)} - p_{10}}{p_{10}^{(A)}} \right) + N_{11} \left(\frac{p_{11}^{(A)} - p_{11}}{p_{11}^{(A)}} \right) = 0$$

$$\frac{\partial L}{\partial \beta_2} = N_{01} \left(\frac{p_{01}^{(A)} - p_{01}}{p_{01}^{(A)}} \right) + N_{11} \left(\frac{p_{11}^{(A)} - p_{11}}{p_{11}^{(A)}} \right) = 0.$$

Now, note that the term $N_{11}\left(\frac{p_{11}^{(A)}-p_{11}}{p_{11}^{(A)}}\right)$ is common to the β_1 and β_2 derivative conditions. Thus, we have that $N_{10}\left(\frac{p_{10}^{(A)}-p_{10}}{p_{10}^{(A)}}\right) = N_{01}\left(\frac{p_{01}^{(A)}-p_{01}}{p_{01}^{(A)}}\right) = -N_{11}\left(\frac{p_{11}^{(A)}-p_{11}}{p_{11}^{(A)}}\right)$. Further, since $N_{lm} > 0$ and $p_{lm}^{(A)} > 0 \forall l, m$, this equality implies two possible cases: 1) $p_{10}^{(A)} \geq p_{10}$, $p_{01}^{(A)} \geq p_{01}$, and $p_{11}^{(A)} \leq p_{11}$; 2) $p_{10}^{(A)} \leq p_{10}$, $p_{01}^{(A)} \leq p_{01}$, and $p_{11}^{(A)} \geq p_{11}$.

Assuming the first case, we then have $B \equiv N_{10}\left(\frac{p_{10}^{(A)}-p_{10}}{p_{10}^{(A)}}\right) = N_{01}\left(\frac{p_{01}^{(A)}-p_{01}}{p_{01}^{(A)}}\right) = -N_{11}\left(\frac{p_{11}^{(A)}-p_{11}}{p_{11}^{(A)}}\right) \geq 0$. Thus, for this case, the derivative condition for β_0 can be re-expressed as: $\frac{\partial L}{\partial \beta_0} = \left(\frac{p_{00}^{(A)}-p_{00}}{p_{00}^{(A)}}\right) +$

$B+B-B=0$, $B\geq 0$. Equality can only be satisfied if the first term is non-positive, which requires $p_{00}^{(A)} \leq p_{00}$. However, since $p_{10} \geq p_{00}$ and $p_{01} \geq p_{00}$, this implies $p_{10}^{(A)} \geq p_{00}^{(A)}$ and $p_{01}^{(A)} \geq p_{00}^{(A)}$.

Next, consider the second case. Suppose that $p_{10}^{(A)} < p_{00}^{(A)}$. This implies that $\beta_1 > 0$, which implies that $p_{11}^{(A)} < p_{10}^{(A)}$. Moreover, $p_{10}^{(A)} \leq p_{10}$. Thus, $p_{11}^{(A)} < p_{10}^{(A)} \leq p_{10} \leq p_{11}$. However, under this case we also have that $p_{11}^{(A)} \geq p_{11}$. Thus, the assumption that $p_{10}^{(A)} < p_{00}^{(A)}$ leads to a contradiction. Applying the same logic, one can show, for this case, that $p_{01}^{(A)} < p_{00}^{(A)}$ also leads to contradiction. Thus, for this case, we must have $p_{10}^{(A)} \geq p_{00}^{(A)}$ and $p_{01}^{(A)} \geq p_{00}^{(A)}$.

Under both possible cases (and thus, in general), we have: $p_{10}^{(A)} \geq p_{00}^{(A)}$ and $p_{01}^{(A)} \geq p_{00}^{(A)}$.
Q.E.D.

Third Result:

$$\begin{aligned} \frac{p_{11}^{(LR')}}{1-p_{11}^{(LR')}} &= e^{\beta_0+\beta_1+\beta_2} = \frac{e^{\beta_0+\beta_1}e^{\beta_0+\beta_2}}{e^{\beta_0}} \\ &= \frac{(p_{10}^{(LR')}/(1-p_{10}^{(LR')}))(p_{01}^{(LR')}/(1-p_{01}^{(LR')}))}{(p_{00}^{(LR')}/(1-p_{00}^{(LR')}))}. \end{aligned} \tag{19}$$

With simple algebra, we obtain:

$$\begin{aligned} 1-p_{11}^{(LR')} &= \frac{(1-p_{01}^{(LR')})(1-p_{10}^{(LR')})p_{00}^{(LR')}}{p_{01}^{(LR')}p_{10}^{(LR')}(1-p_{00}^{(LR')})}p_{11}^{(LR')} \\ &= \frac{(1-p_{01}^{(A)})(1-p_{10}^{(A)})p_{00}^{(A)}}{p_{01}^{(A)}p_{10}^{(A)}(1-p_{00}^{(A)})}p_{11}^{(LR')}. \end{aligned} \tag{20}$$

Solving for $p_{11}^{(LR')}$, we then obtain:

$$p_{11}^{(LR')} = \frac{p_{01}^{(A)}p_{10}^{(A)}(1-p_{00}^{(A)})}{p_{00}^{(A)} - p_{01}^{(A)}p_{00}^{(A)} - p_{10}^{(A)}p_{00}^{(A)} + p_{01}^{(A)}p_{10}^{(A)}}. \tag{21}$$

Correspondingly, for the maximum likelihood model of AIM form $\log(1-P(C=1|X_1,X_2))) = \beta'_0 + \beta'_1 X_1 + \beta'_2 X_2$, we have $\beta'_0 = \log(1-p_{00}^{(A)})$, $\beta'_1 = \log(\frac{1-p_{10}^{(A)}}{1-p_{00}^{(A)}})$, and $\beta'_2 = \log(\frac{1-p_{01}^{(A)}}{1-p_{00}^{(A)}})$. Thus,

$$\begin{aligned} 1-p_{11}^{(A)} = e^{\beta'_0+\beta'_1+\beta'_2} = \frac{(1-p_{10}^{(A)})(1-p_{01}^{(A)})}{(1-p_{00}^{(A)})} \text{ and} \\ p_{11}^{(A)} = 1 - \frac{(1-p_{10}^{(A)})(1-p_{01}^{(A)})}{(1-p_{00}^{(A)})}. \end{aligned} \tag{22}$$

Now, let us check the sign of $p_{11}^{(LR')} - p_{11}^{(A)}$. We can write:

$$\begin{aligned} p_{11}^{(LR')} - p_{11}^{(A)} &= (1-p_{11}^{(LR')})\left(\frac{(1-p_{11}^{(A)})}{(1-p_{11}^{(LR')})} - 1\right) \\ &= (1-p_{11}^{(LR')})\left(\frac{p_{01}^{(A)}p_{10}^{(A)}}{p_{00}^{(A)}p_{11}^{(LR')}} - 1\right), \end{aligned} \tag{23}$$

where the latter expression is obtained using (20) and (22).

We now compare $\frac{p_{01}^{(A)}p_{10}^{(A)}}{p_{00}^{(A)}p_{11}^{(LR')}}$ to 1. First, using (21), we have:

$$\begin{aligned}\frac{p_{01}^{(A)}p_{10}^{(A)}}{p_{00}^{(A)}p_{11}^{(LR')}} &= \frac{p_{00}^{(A)} - p_{00}^{(A)}p_{01}^{(A)} - p_{00}^{(A)}p_{10}^{(A)} + p_{01}^{(A)}p_{10}^{(A)}}{p_{00}^{(A)}(1-p_{00}^{(A)})} \\ &= \frac{p_{01}^{(A)}(p_{10}^{(A)} - p_{00}^{(A)}) + p_{00}^{(A)} - p_{00}^{(A)}p_{10}^{(A)}}{p_{00}^{(A)}(1-p_{00}^{(A)})} \\ &= \frac{(p_{01}^{(A)} - p_{00}^{(A)})(p_{10}^{(A)} - p_{00}^{(A)}) + p_{00}^{(A)}(p_{10}^{(A)} - p_{00}^{(A)}) + p_{00}^{(A)} - p_{00}^{(A)}p_{10}^{(A)}}{p_{00}^{(A)}(1-p_{00}^{(A)})} \\ &= \frac{(p_{01}^{(A)} - p_{00}^{(A)})(p_{10}^{(A)} - p_{00}^{(A)}) + p_{00}^{(A)}(1-p_{00}^{(A)})}{p_{00}^{(A)}(1-p_{00}^{(A)})}.\end{aligned} \tag{24}$$

Now, since $(p_{01}^{(A)} - p_{00}^{(A)}) \geq 0$ and $(p_{10}^{(A)} - p_{00}^{(A)}) \geq 0$, the final expression must also be greater than or equal to 1. Thus, we have $\frac{p_{01}^{(A)}p_{10}^{(A)}}{p_{00}^{(A)}p_{11}^{(LR')}} \geq 1$. Now, examining (23) and noting that $1 - p_{11}^{(LR')} > 0$, we have finally proved that $p_{11}^{(LR')} \geq p_{11}^{(A)}$. Furthermore, examining $(p_{01}^{(A)} - p_{00}^{(A)})(p_{10}^{(A)} - p_{00}^{(A)})$ in the final expression in (24), it is seen that this expression equals 1, and, thus, $p_{11}^{(LR')} = p_{11}^{(A)}$, if and only if $p_{01}^{(A)} = p_{00}^{(A)}$ or $p_{10}^{(A)} = p_{00}^{(A)}$.

Q.E.D.

C.7.5 Proof of Lemma 5.2:

$$\begin{aligned}L' - L &= N\sum_{m=1}^{M} p_m \log(q'_m) - N\sum_{m=1}^{M} p_m \log(q_m) \\ &= N\sum_{m=1}^{M} p_m \log(\lambda p_m + (1-\lambda)q_m) - N\sum_{m=1}^{M} p_m \log(q_m) \\ &\geq \sum_{m=1}^{M} (\lambda p_m \log(p_m) + (1-\lambda)p_m \log(q_m) - \sum_{m=1}^{M} p_m \log(q_m) \\ &= \lambda\sum_{m=1}^{M} p_m \log(\frac{p_m}{q_m}) = \lambda D_{\mathrm{KL}}(\mathcal{P}||\mathcal{Q}) \geq 0.\end{aligned} \tag{25}$$

Here, the first inequality is obtained from Jensen's inequality applied to the logarithm function, and $D_{\mathrm{KL}}(\mathcal{P}||\mathcal{Q})$ is the Kullback-Leibler distance between pmfs (which is non-negative). Thus, $L' \geq L$. Note that equality is achieved if $\lambda = 0$, in which case $q'_m = q_m \forall m$. Moreover, since the log() is strictly concave, again by Jensen's inequality, equality is *only* possible for $\lambda = 0$ or 1; in this case, only if $q'_m = q_m \forall m$.

Q.E.D.

[Note that while Theorem 5 only considers two factors (those involved in a possible interaction), our experimental results are consistent with this theorem holding more generally, when there are

an arbitrary number of factors and one wishes to test for interaction between any pair of them while accounting for effects from all of them.]

C.8 Applicability for non-binary factors

Both in the above derivation of AIM and in developing its theoretical properties, we assumed that factors are binary. All of the above results can be straightforwardly extended for the case where factors are non-binary but categorical. In particular, a nonbinary categorical variable X with cardinality L can be recoded as a vector of L binary factors $\in \{0,1\}$, with only one of these factors "on" to specify a value for X. The AIM model can also be *applied* when the variables X_i are quantitative (or ordinal). However, the AIM model form is not logically derivable in the same way as given above for binary factors. Moreover, while AIM's invariance properties hold for nonbinary and quantitative factors (since we do not assume factors are binary in the proofs of Theorems 2,3, and 4), the rigorous proof of Theorem 5 on detection power relies on the assumption of binary factors.

Appendix D: Evaluation of Type I Error

To test whether our AIM approach can detect interactions at the right significance levels, we assessed the type 1 error via simulation studies under the null hypothesis (Methods). Accuracy of type 1 error is crucial for any hypothesis testing methods that detect interactions based on their p-values, because if the type 1 error is either too conservative or too liberal, the p-value loses its intended meaning and fails to reflect the actual false positives. Using the simulation data with varying case fraction, we will show that for all scenarios the empirical type 1 error produced by AIM closely approximates the expected type 1 error. We also show that the Q-Q plot closely aligns with the diagonal line with no noticeable deviation(Fig. S1), even when the factors are correlated or imbalanced.

D.1 Varying case fractions

Figure S2 shows the empirical type I error for AIM (evaluated when the null hypothesis of no interaction is valid) at significance level 0.05 for varying case fraction. The gray region is the 95% confidence interval. We assessed the influence of the case fraction on the empirical type I error. Each estimate is based on 1000 tests, with the empirical type I error calculated as the ratio between

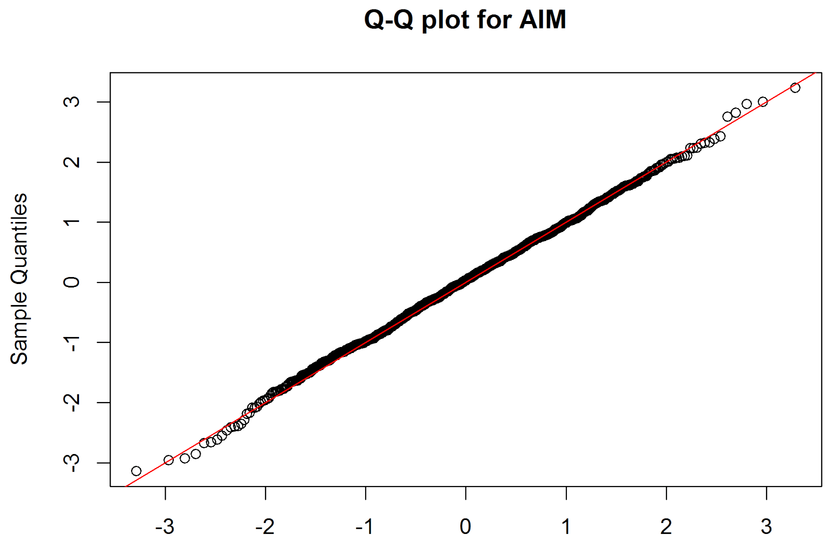

Figure S1: Q-Q plot for AIM.

the number of tests that have p-value smaller than 0.05 and the total number of tests. The AIM model used to generate the data here is: $\log(1-P(C=1|\underline{x})) = a(-0.337-0.336x_1-0.336x_2)$, with a varied to sweep the range of case fractions from 0.05 to 0.95. We can see that for all scenarios the empirical type I error closely approximates the expected type I error.

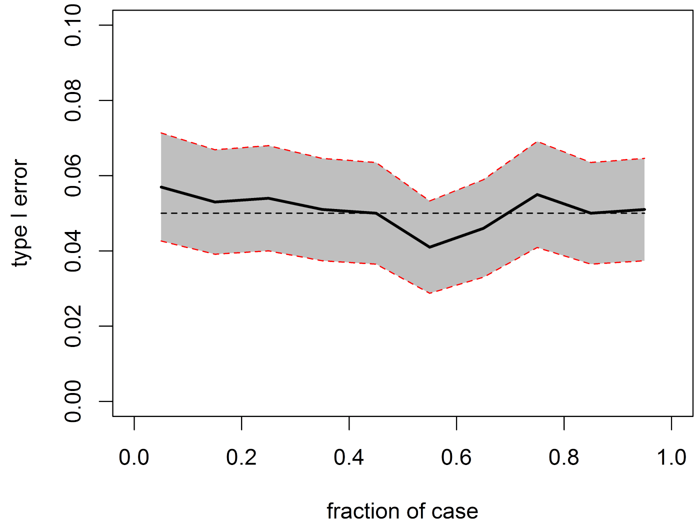

Figure S2: Empirical type I error of AIM with varying case fraction.

D.2 Missing factors

Figures S3.a, S3.b, and S3.c show a comparison of the empirical type I error rate at significance level 0.05 for LR and AIM for the case of missing factors. For each comparison, the left (dark) bar is LR's measure and the right (light) bar is AIM's. This is the convention used in all our type 1 error figures. For AIM experiments, the data was generated according to the baseline AIM model, and for LR experiments, the data was generated according to the baseline LR model, with the ground-truth AIM and LR model parameter values chosen so that the marginal effects of each of the factors were the same for the two (AIM and LR) baseline models. Because the marginal effects are the same, it is reasonable to compare the AIM and LR type 1 errors in the same plot, for each scenario. We simulated in total 18 scenarios with different case fractions and number of missing factors. To get reliable estimates of type I error, 10,000 datasets were simulated for each scenario. Both the point estimate and the 95% confidence interval are shown in the figure. All the scenarios are designed to have marginal effects with an odds ratio of 2 for the observable factors. In Figure S3.a, we simulated one missing factor with effect size of 15. In Figure S3.b, we simulated 10 missing factors with comparable effect sizes as for the observable factors. In Figure S3.c we simulated 100 missing factors with effect sizes of 1.1. In all scenarios, the empirical type I error rates for AIM match the theoretical value (0.05) very well. The inflation of type I error rate for LR has multiple causes. However, generally speaking, the fewer number and the larger effect sizes of missing factors result in larger inflation. Also seen from the figure, larger inflation occurs when the case-control ratio deviates from balanced (0.5). Interestingly, we observed no inflation for LR when the case fraction is 0.5.

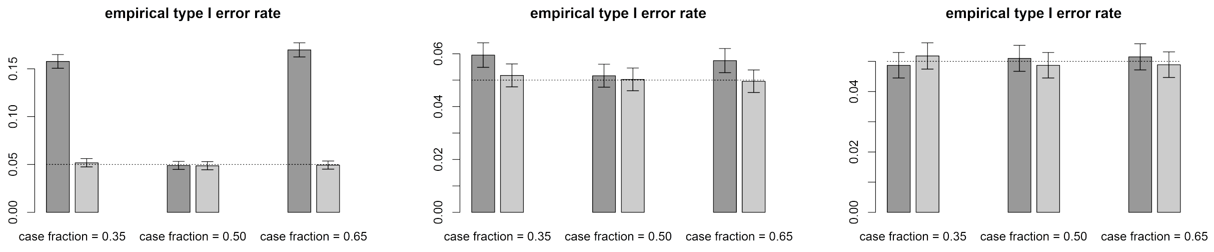

(a) Few missing factors (b) Moderate missing factors (c) Many missing factors

Figure S3: Empirical type I error rate at significance level 0.05 for LR (dark grey) and AIM (light grey) when there are a) a few missing factors with large effect size; b) a moderate number of missing factors with moderate effect size; and c) when there are a lot of missing factors with small effect size.

D.3 Surrogate markers

Figures S4.a and S4.b compare the empirical type I error rates for both LR and AIM when the observed markers are surrogate instead of causal. Each empirical type I error rate is estimated based on 10000 experiments. The dashed lines indicate the expected type I error rate and the 95% confidence intervals for each estimate are marked by the corresponding error bars. In Figure S4.a, the effect size for the observable surrogate markers is small and approximately 1.5 in terms of odds ratio. In Figure S4.b, the effect size is around 5. The empirical type I error for AIM is close to the expected in all cases. The accuracy of type I error rate for LR is dependent on two factors – the effect size and the degree of correlation between a surrogate and its associated causal factor (r^2). Larger effect size and weaker correlation generally imply larger deviation from the expected value.

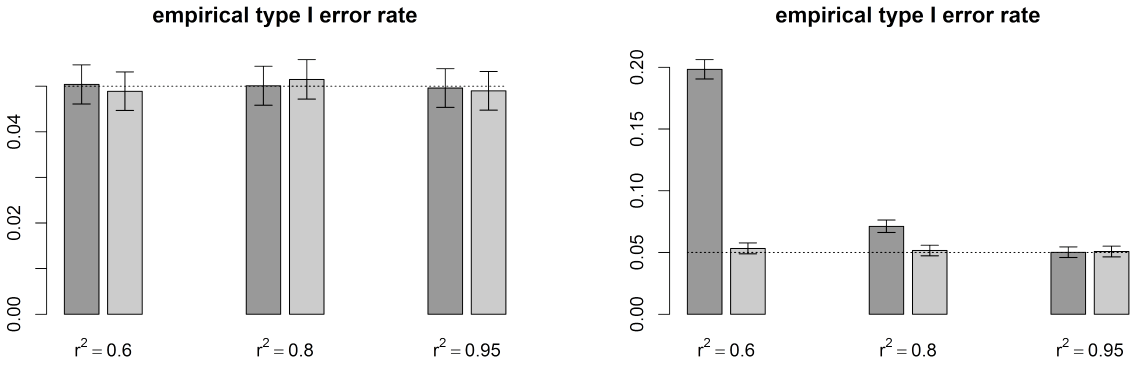

(a) Weak marginal effects

(b) Strong marginal effects

Figure S4: Empirical type I error rate at significance level 0.05 for LR (dark grey) and AIM (light grey) when the surrogate markers have a) weak marginal effects and b) strong marginal effects; r^2 is the correlation between a surrogate factor and its associated causal factor.

D.4 Subtypes

Figures S5.a and S5.b show the empirical type I errors when there are subtypes. We simulated four scenarios with different effect sizes and number of subtypes. Here we assume both risk factors have the same marginal effect size. When the effect size is large, the overall distribution deviates from the null LR model significantly, though each subtype follows the LR model. Each subtype independently generates the status of 'case' or 'control', with the overall status a 'case' if any subtype status is a 'case'. We also notice that when the effect size is weak (odds ratio = 1.5), the

effect of the subtypes is negligible.

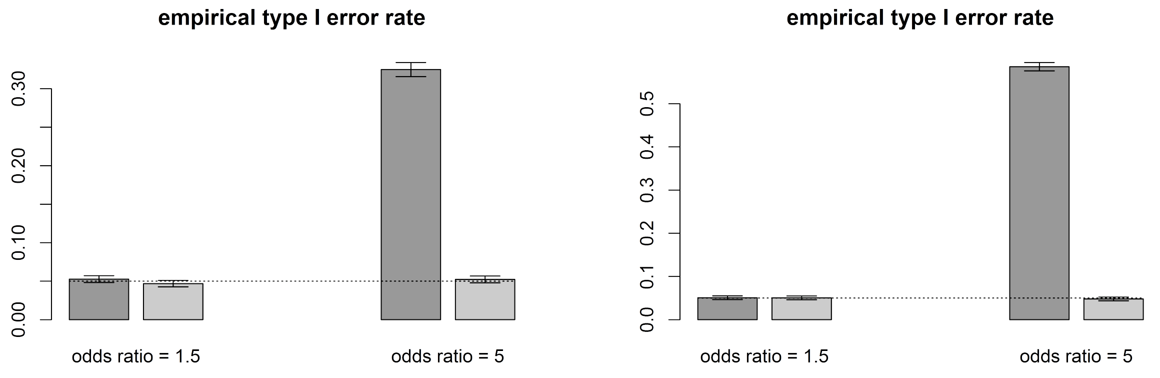

(a) 3 subtypes

(b) 20 subtypes

Figure S5: Empirical type I error rate at significance level 0.05 for LR (dark grey) and AIM (light grey) when there are a) 3 subtypes and b) 20 subtypes. Odds ratio refers to the marginal effect.

D.5 Impact of the violation of the independence assumption in Theorems 2 and 3

The simulations in Subsections D.2 and D.3 confirm Theorems 2 and 3 under independence between the observable factors (when missing factors exist) or the causal factors (when surrogate factors are observed). These assumptions are non-trivial. Here, we investigate numerically the scenarios where the independence assumptions are violated. Our experiments show different effects of the violation of the assumption for the two theorems. Though we cannot prove it theoretically, it seems that Theorem 2 still practically holds even when the observed factors are strongly correlated. On the other hand, we do observe that the violation of the independence assumption leads to a systematic, though often small, breakdown of Theorem 3. (Note that Theorem 4 does not make an independence assumption.)

To test the impact of the violation of the independence assumption when there are missing factors, we tried various settings as in section D.2. We do not see obvious inflation of the type I error for AIM. Based on our experience with LR, we hypothesized that the setting of a few but large-effect missing factors is most likely to demonstrate an impact on AIM. The left subfigure in Figure S6 shows the empirical type I error rate under this setting. Using an interval of 0.1, we surveyed the correlation with coefficients ranging from -0.9 to 0.9. For all 19 sets of experiments, all but r=0.2 have the expected type I error rate falling in the 95% confidence interval. However,

the only exception should also be expected due to the effect of multiple tests. In fact, we fixed r=0.2 and conducted the experiment again. This time, the expected type 1 error rate did fall within the 95% confidence interval. We tried multiple settings as in section D.3 to test the impact of the violation on the type I error rate when surrogate factors are measured. Although for the majority of the settings the effect is not detectable, a small fraction of settings consistently show inflated type I error. The right figure of Figure S6 illustrates the effect under a typical scenario. The correlation is 0.8 between the causal and surrogate factors. The inflation becomes obvious when the correlation between the two causal factors is very strong. We do not see observable inflation for moderate correlations. To further test whether the inflation is due to some random effect, we fixed the correlation at 0.9 and increased the number of simulations to 100,000 to narrow the confidence interval of the estimated type I error rate. We got a point estimate as 0.0615 and the 95% confidence interval is [0.0599, 0.0630].

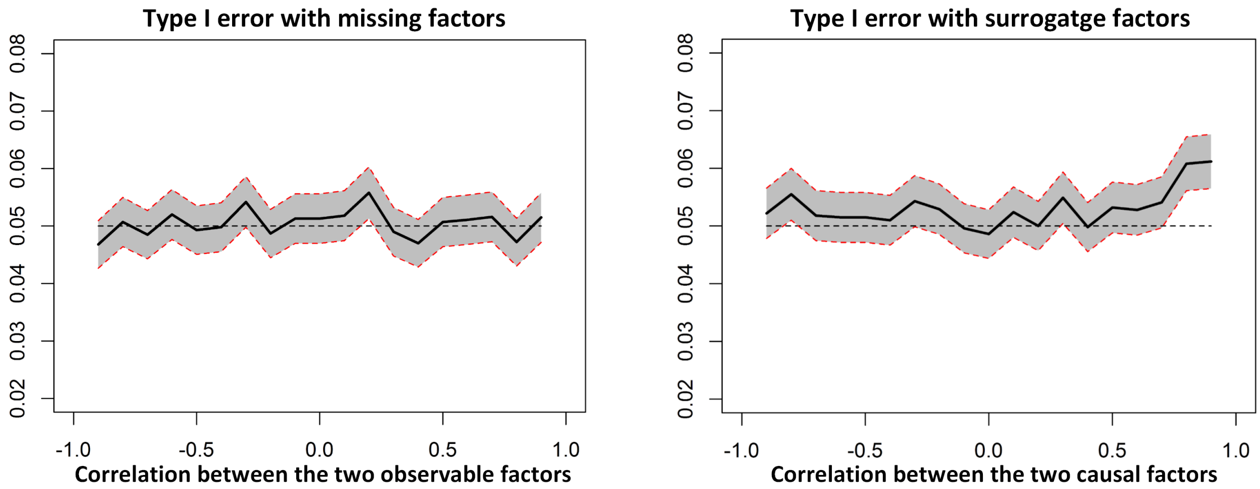

Figure S6: Empirical type I error rate at significance level 0.05 for AIM when the independence assumptions in Theorems 2 and 3 are violated. The left and right figures demonstrate the scenarios for Theorem 2 (missing factors) and Theorem 3 (surrogate factors), respectively. Red-dashed lines indicate the 95 % confidence interval.

Appendix E: More Simulation Results on Power

We further perform extensive simulation studies to evaluate the detection power of AIM and LR in a controlled setting, under varying parameter settings which characterize the population being studied, as well as under the three confounding scenarios prominently identified in this paper – missing factors, surrogate factors, and disease subtypes. The goal of these studies is to understand

the effect on power of different parameter settings and of these scenarios on both models. Uniformly across these extensive simulation studies, AIM is found to be substantially more powerful than LR.

In this section we report results on various synthetic datasets to assess the performance of AIM in detecting true interaction effects. A comprehensive set of scenarios were simulated to evaluate how the power of AIM is affected by sample size, effect size, case-control ratio, risk factor allele frequency, p-value threshold, main effects, correlation between risk factors, missing factors, surrogate factors, and disease subtypes. For every experiment, the performance of LR was also evaluated, with special attention paid to the different trends observed for AIM and LR. In all of the reported experiments, power was empirically estimated based on 1000 random simulations. When we investigated the effect of one parameter, we fixed all other parameters. The default p-value threshold (alpha value) was set as 0.05. By default, we assumed risk factors are independent. In most of the experiments, the ground-truth interaction models were based on an LR model with non-zero interaction terms, as follows: $\log(\frac{p}{1-p}) = \alpha_0 + \alpha_1 x_1 + \alpha_2 x_2 + \alpha_3 x_1 x_2$. Note again that this means the LR approach is *matched* to the ground-truth interaction model, whereas AIM is not – even so, AIM is guaranteed to be more powerful to detect synergistic interactions. Here, both risk factors are binary. The odds ratio is used to represent the effect size. Thus, e^{α_1} and e^{α_2} are the two main effect sizes and e^{α_3} the interaction effect size. By default, we set the main effect size for both risk factors to 1.5. The interaction effect size was also set to 1.5. The risk allele frequency, that is, the frequency of $x_i = 1, i = 1, 2$ was set to 50%; α_0 was adjusted so that the case fraction was around 50%. We had two different default settings for the sample size. When the experiment assesses the impact of interaction effect size, the sample size was set to 1000; otherwise the sample size was set to 2000. The smaller sample size was designed to survey the impact of a larger range of interaction effect sizes. The above-described interaction model was also extended to include high-order interactions and more complex interaction forms. We took advantage of existing interaction models[13] and applied both AIM and LR to them. Specifically, five more interaction models were tested, spanning from 2-way to 5-way interactions and involving ternary variables. In the following subsections we give detailed discussion on the results for each set of experiments; finally we summarize our overall conclusions.

E.1 Impact of sample size and effect size

Figures S7.a, b, and c show how power is affected by sample size with the interaction effect size fixed at 1.1, 1.5, and 3, respectively. Figure S8 shows how power is affected by effect size with the

sample size fixed at 1000. As expected, for both methods the power increases from 0 to 1 when either the sample size or the effect size is increased. We can also see that the effect size has more dramatic impact on power than the sample size. For AIM, 1600 samples are needed to achieve 80% power when the effect size is 1.5, compared to 280 samples when the effect size is doubled to 3.0. Under all scenarios AIM is always better powered than LR. However, the difference in power is dependent on the effect size. For example, in Figure S7.a, to achieve 80% power, 13,000 and 57,000 samples are needed for AIM and LR, respectively. In Figure S7.b, when the effect size is 1.5, to achieve 80% power 1,600 and 3,200 samples are needed for AIM and LR, respectively. Generally speaking, the relative gain of AIM over LR is larger for smaller effect sizes and for (relatively) small sample sizes at a given effect size, as seen in Figures S7.a, b, and c. This is encouraging because one often faces problems with small effect size (and limited sample size) in real applications. However, we can also observe that the maximum gain in power of AIM over LR over the range of sample sizes (as well as the area between the two power curves) decreases as the effect size increases.

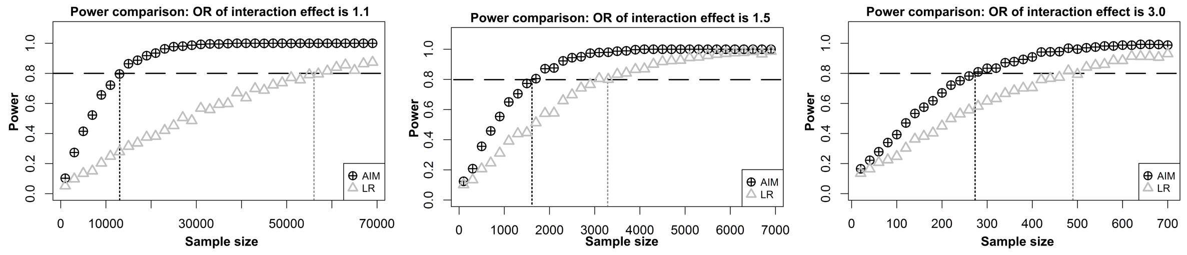

(a) Small interaction effect size (b) Moderate interaction effect size (c) Large interaction effect size

Figure S7: Power vs sample size when the interaction effect size is fixed at a) an odds ratio of 1.1; b) an odds ratio of 1.5; c) an odds ratio of 3. The case fraction was 50% and the main effect size was 1.5 for both risk factors.

E.2 Impact of case-control ratio

Figure 2c illustrates how the case-control ratio influences the power and how the two methods differ. We surveyed the fraction of cases from the very low end (0.1) to the very high end (0.9). It should be expected that power will not achieve its maximum at either end. Indeed, when the cohort is composed of all cases or controls, there is no way to evaluate the difference between cases and controls. Thus, the maximum power should be achieved at some intermediate value. Recalling that LR is a symmetric with respect to case/control status, it is reassuring to see that LR gets maximum power at a case fraction of 0.5. A striking difference here is that AIM achieves its maximum power when the fraction of cases is around 0.3. We can also observe that the gain of AIM over LR is

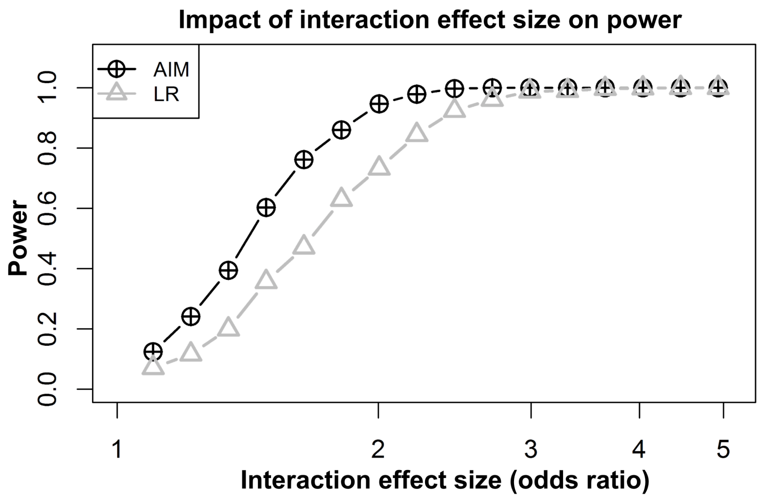

Figure S8: Power versus interaction effect size. The sample size is fixed at 1000. The main effect sizes for both risk factors were set at the odds ratio of 1.5. The case fraction is fixed at 50%.

larger when the fraction of cases is smaller. This phenomenon is rooted in the defining formulas for AIM and LR. Comparing the AIM form $\log(1-p)$ to the LR form $\log(p)-\log(1-p)$, where p is the probability of a case, we can see that, neglecting the sign, the two forms get close when p approaches 1.

E.3 Impact of risk allele frequency

Figure 2d shows how the risk allele frequency impacts power. The allele frequencies were simulated to range from 0.1 to 0.9. The power is decreased for both AIM and LR when the risk allele frequency is either extremely low or high. The power for LR is symmetric with respect to the risk allele frequency, achieving its maximum at 0.5. The power curve for AIM is skewed to the higher frequencies – when the risk allele frequency is 0.1, AIM has power of 0.4; when the risk allele frequency is 0.9, the power is 0.55. Considering that we used 1000 simulations to calculate power, this difference is highly unlikely to be due to random fluctuations. We do not have an analytical characterization of how the risk allele frequency asymmetrically affects AIM's power, with the higher allele frequencies more power-favorable. However, we believe this is again a consequence of the asymmetry of AIM with respect to case/control status.

E.4 Impact of main effect size

Figure 2f demonstrates how the main effect size affects the power to detect an interaction when the interaction effect size is fixed. We observe that AIM and LR follow very different trends. LR's

power slightly decreases as the main effect size increases. The power of LR is 0.352 at a main effect size of 1.1 and reduces to 0.279 at a main effect size of 5.0. This decline in power is even more apparent for larger main effect sizes. Although not shown in Figure 2f, we observed that LR's power falls to 0.16 for a main effect size of 20. These results are not surprising – with the interaction effect size fixed, the increase in main effect size increases the variance of the estimate of interaction effect size. On the other hand, AIM's power increases as the main effect size is increased. This can be understood by recognizing i) that the interaction effect is the difference between the true effect and the one predicted by the null hypothesis; and ii) that AIM and LR posit very different null hypotheses. In particular, in this experiment the data were generated based on an LR model, with the interaction effect size fixed while varying the main effect size. A fixed interaction effect for an LR model will almost assuredly give a *variable* interaction effect size for the AIM model, as the main effect size is varied.

E.5 Impact of p-value threshold

Figure 2g shows how the sample size needed for 80% power is dependent on the p-value threshold of significance. We varied the p-value threshold from 0.05 to 5e-8. It appears that the sample size is linearly proportional to the log-transformation of the p-value threshold, for both AIM and LR. However, AIM's slope is smaller than that of LR. When the p-value threshold is 0.05, 1600 and 3200 samples are needed for AIM and LR, respectively, *i.e.*, twice AIM's number of samples are needed for LR. When the p-value threshold is 5e-8, the ratio is 2.17 (computed as 165,000 divided by 76,000).

E.6 Impact of correlation between risk factors

Figure 2e illustrates how the correlation between the two risk factors affects power. High absolute correlation significantly reduces power. For example, when the correlation is -0.95, the power for AIM reduces from 0.902 to 0.282, while the power for LR reduces from 0.626 to 0.153. The maximum power is achieved for both AIM and LR when the two risk factors are independent. It is worth noting that unlike for the case-control ratio and allele frequency, AIM's power, similar to LR's, exhibits symmetric dependence on the correlation between the two factors.

E.7 Impact of missing risk factors

Figure S9.a compares the power for AIM under the three scenarios of no missing factors, a few strong missing factors, and many weak missing factors. A similar comparison for LR is shown in Figure S9.b. We simulated three strong and one hundred weak missing factors, but with the overall effect designed to be the same.

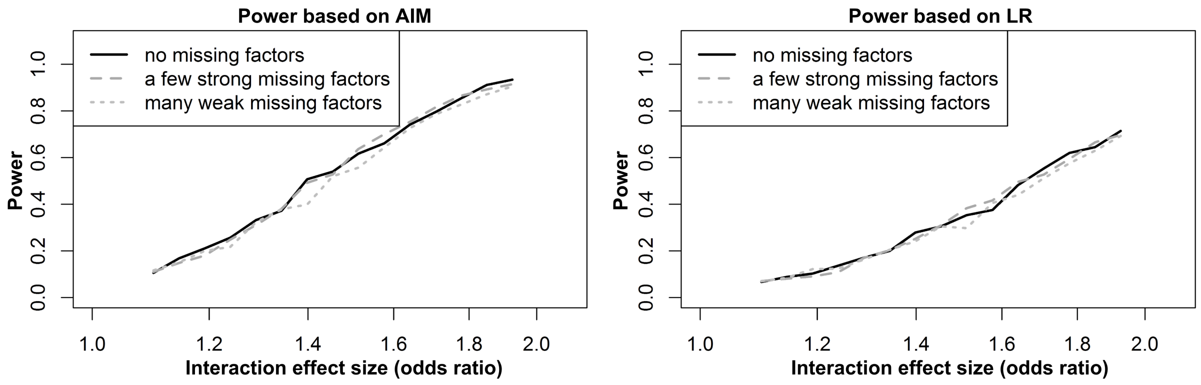

(a) AIM under missing factors

(b) LR under missing factors

Figure S9: Power estimated based on 1000 simulation data sets when there are no missing factors, a few strong missing factors, or many weak missing factors for a) AIM and b) LR.

From these figures we can see that the power does not change too much for both AIM and LR when there are missing factors. The change is so small that we cannot draw definitive conclusions from the two figures; however, the existence of a few strong missing factors does appear to decrease the power. To further assess this, we simulated 10,000 datasets, focusing on an interaction effect size of 1.5, with the results shown in Table S3.

	No missing factors	A few strong missing factors	Many weak missing factors
AIM power	0.602 [0.592,0.611]	0.555 [0.545, 0.565]	0.604 [0.594, 0.614]
LR power	0.348 [0.338, 0.357]	0.317 [0.308,0.326]	0.347 [0.337, 0.356]

Table S3. Power comparison when there are missing factors. The interaction effect size was fixed at 1.5. Power was estimated based on 10000 simulations. Confidence intervals (shown in brackets) were computed using a binomial distribution.

The existence of a few strong missing factors indeed decreases power for both AIM and LR; however, many weak missing factors did not have any observable effect on the power. Since missing risk factors do not change the power much, it is expected that AIM will still be more powerful than

LR, which is confirmed by Figures S10.a and b.

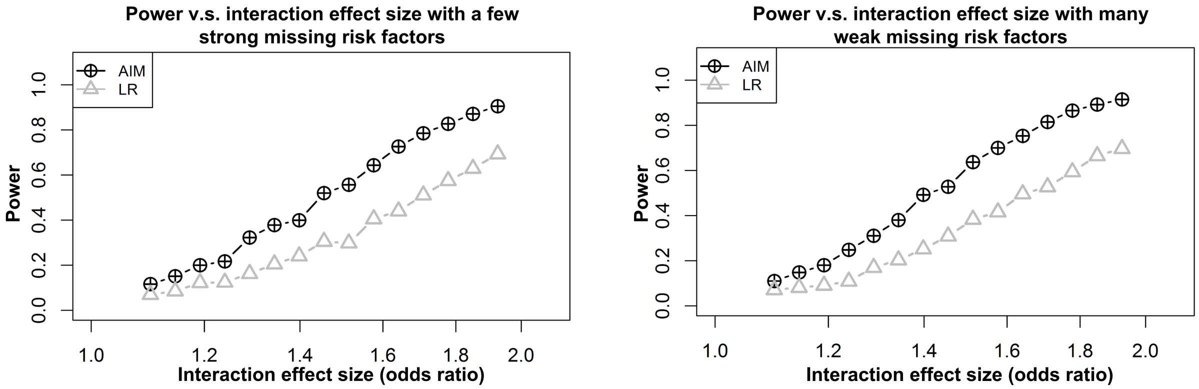

(a) Several strong factors missing

(b) Many weak factors missing

Figure S10: Interaction detection power versus interaction odds ratio for AIM and LR models when a) several strong non-interacting factors are missing and b) many weak non-interacting factors are missing.

E.8 Impact of surrogate factors

Figure S11 shows how power is affected when surrogate rather than causal risk factors are measured. Surrogate factors have very large impact on the power for both AIM and LR. For example, the power for AIM drops from 0.877 to 0.486 when surrogate factors with correlation coefficient of 0.8 to their causal counterparts are observed. Similarly, the power for LR drops from 0.607 to 0.278. No matter how great the power decreases, we see that AIM's power is always greater than LR's. It is also noteworthy that both AIM and LR are symmetric with respect to the correlation coefficient between surrogate and causal factors.

E.9 Impact of subtypes

Figures S12.a and b illustrate how the existence of subtypes impacts the power for both AIM and LR. When there are subtypes, the power for both AIM and LR are significantly increased. It seems that the existence of subtypes make the interaction effect stronger. Even so, we see in Figure S13 that, with subtypes, AIM has better power to detect the interaction effect than LR.

E.10 Power for an antagonistic interaction

Figure S14 shows how the power varies with interaction effect size for an interaction that is antagonistic. For a ground-truth LR model with interaction terms, an antagonistic interaction is defined

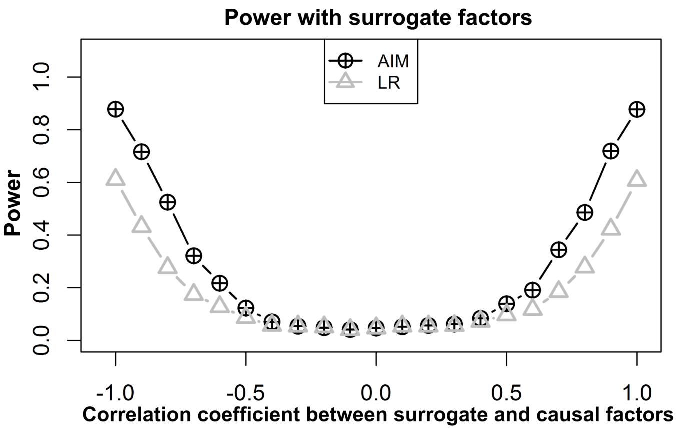

Figure S11: Interaction detection power under the surrogate factors scenario, as a function of the correlation between surrogate and causal factors, for AIM and LR models.

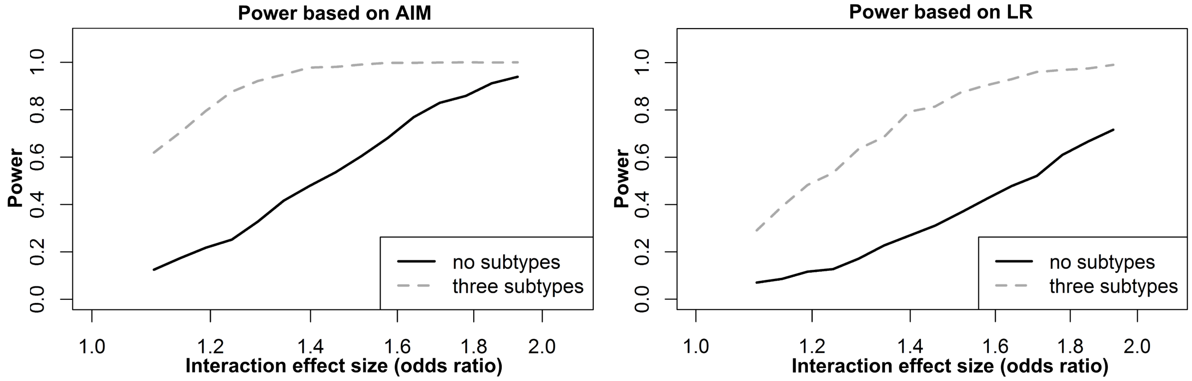

(a) AIM under subtypes

(b) LR under subtypes

Figure S12: Interaction detection power versus interaction odds ratio for a) AIM and b) LR models under the cases of no disease subtypes and three disease subtypes.

as one with an interaction coefficient α_3 that is negative. Equivalently, it is such that the effect size (odds ratio) is smaller than one. The experiments were conducted exactly as in Figure S7 except that the effect size here was set to the reciprocal of the value used to produce Figure S7. The experimental results are consistent with the statement in our theorem, with the power for LR larger than that for AIM when the interaction is antagonistic. However, the reduction in power for AIM relative to LR is quite small. Interestingly, we observe another asymmetric characteristic of AIM through this experiment – LR's power is symmetric with respect to the log-transformation of the odds ratio, while AIM's is not. Indeed, the gain of AIM over LR for synergistic interactions is much larger than the loss relative to LR for antagonistic ones.

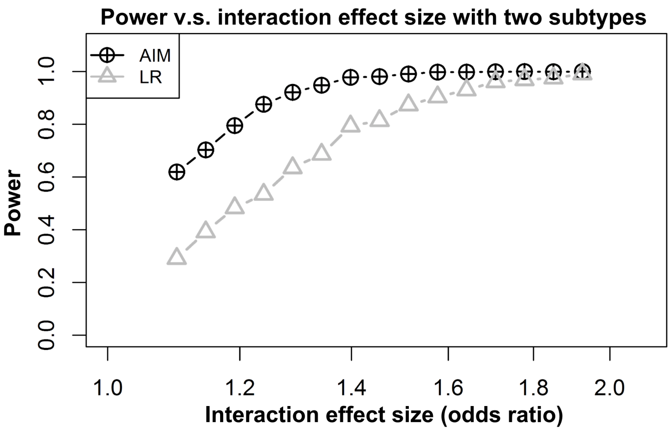

Figure S13: Interaction detection power when there are two disease subtypes, as a function of interaction odds ratio, for AIM and LR models.

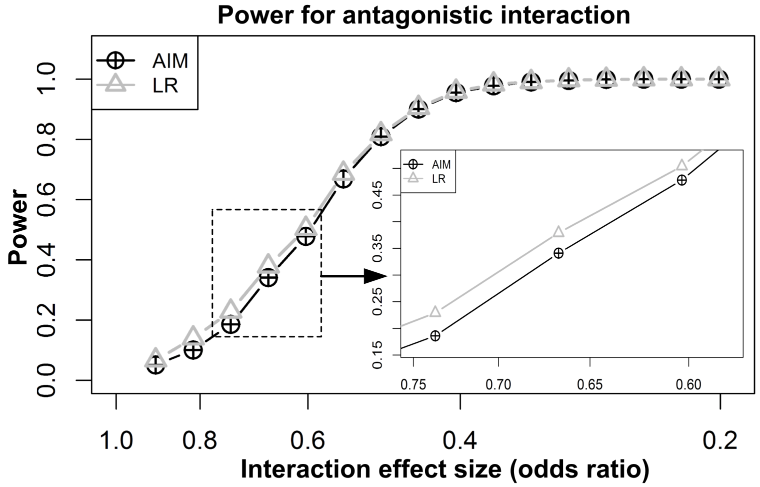

Figure S14: Power of AIM and LR versus interaction effect size for an antagonistic interaction.

E.11 Experiments on five previous published interaction models

The simulated population retained the basic patterns of linkage disequilibrium, missing data, and allele frequencies observed in the original genome scan data. Multiple interactions (five) simultaneously exist in the simulated population (reasonable, considering complex disease mechanisms) and jointly decide the phenotype for each individual. The five interaction models vary in interaction order (from two-way up to five-way), genetic models (dominant, recessive, or additive), incomplete/complete penetrance, minor allele frequency, and marginal effects size. The chosen models[14], specified in reference 14, were motivated by complex genetic traits (such as autoimmune diseases, diabetes, and arthritis) where there are multiple loci contributing to disease risk and where there are both some relatively large interaction effects as well as more modest ones[14]. We considered

data sets with 1000 samples and 100 SNP variables. Fifteen of these SNPs participate in the five interaction models, with the remaining SNPs having no ground-truth association with the disease status.

Figure 2h shows the statistical significance of each ground-truth interaction model, as detected by the AIM and LR models. Note that AIM achieves smaller p-values for all five models. Smaller p-values imply fewer data subjects are needed to detect an interaction at a minimum required level of significance.

E.12 Overall conclusions from power comparisons

Summarizing the previous discussions, we have the following overall conclusions on the power comparisons: (1) For synergistic interactions, under all scenarios and parameter combinations, AIM is always better powered than LR. (2) The power gain of AIM over LR is larger when the interaction effect size is small and/or when the sample size is relatively small. (3) The power gain of AIM over LR is larger when the main effect is large. (4) The power gain of AIM over LR is larger for small case fractions, as opposed to large case fractions. (5) The existence of missing risk factors, surrogate factors or subtypes may decrease or increase the power for both AIM and LR, but AIM always has larger power than LR. (6) AIM is not only mathematically asymmetric (with respect to disease status) – its power is also asymmetric, with respect to both the case-control ratio and the allele frequency.

Appendix F: More details on Real Application

F.1 Application of AIM on real venous thrombosis dataset detects interaction between variants of factor V and prothrombin contributing to increased risk of venous thrombosis

In the Legnani et al. study[15] (Table 1), the odds ratio associated with the use of oral contraceptive but no thrombophilic genetic risk mutation is 1.95, and the odds ratio associated with genetic defects but no use of contraceptive is 4.79. According to the multiplicative model, the odds ratio associated with the presence of both risk factors should be 9.34, while the observed odds ratio is 27.4. This is strong evidence of interaction. Indeed, by applying LR, we get a p-value of 0.021, which is statistically significant. If we apply AIM, we get a p-value of 0.00062. There are 947 subjects in the Legnani et al. study. When all the frequencies of the risk factors and the effect size

are kept the same, we estimate that, to achieve the 0.05 significance level, LR requires 676 subjects, while AIM needs only 303 subjects.

For the Martinelli et al. study[16] (Table S4), the odds ratio associated with the presence of both risk factors (according to a multiplicative model) is expected to be 11.9, compared to the observed value of 18.1. Both studies have the same effect direction, that is, the observed odds ratio is larger than the expectation. Due to the limited sample size, the conclusion is not statistically significant in the Martinelli et al. study. The p-value generated by LR is 0.618 and the p-value obtained from AIM is 0.183. To achieve the 0.05 significance level, the estimated sample size associated with LR is 4391, while AIM requires just 614 subjects.

thrombophilic genetic risk mutation	oral contraceptive	controls	cases	odds ratio
-	-	127	35	1
-	+	41	52	4.60
+	-	7	5	2.59
+	+	4	20	18.1

Table S4. Martinelli et al. study: risk of venous thrombosis according to the presence of thrombophilic genetic mutation and the use of oral contraceptive.

F.2 Application of AIM on real esophageal cancer dataset detects smoking-alcohol interaction contributing to increased risk of esophageal cancer

It has long been suggested that tobacco smoking and alcohol consumption interplay to influence the risk of cancer[17]. Alcohol may act as a cocarcinogen and enhance the carcinogenic effects of other chemicals from tobacco smoking. Indeed, quite a few epidemiological studies have confirmed their interaction effect on esophageal cancer[18,19]. The Castellsague et al. study[18] is probably the first large scale case-control study implying the interaction effect of tobacco smoking and alcohol consumption on esophageal cancer.

F.3 Application of AIM on real bladder cancer dataset detects NAT2-smoking interaction contributing to increased risk of bladder cancer

With hundreds of thousands of new cases diagnosed each year worldwide, bladder cancer is increasingly important for public health, with tobacco smoking the predominant known risk factor. In Europe, smoking is estimated to cause over half of bladder cancer cases in men and one-third of

cases among women[20]. Multiple carcinogens have been found in tobacco smoke, including polycyclic aromatic hydrocarbons, N-nitrosamines, aromatic amines, heterocyclic amines, and aldehydes. Originally inert, these carcinogens may undergo both activation and detoxification. Imbalance between activation and detoxification will increase the bladder cancer risk through accumulation of active carcinogen metabolites and increased DNA adduct formation[21].

The NAT2 gene encodes an enzyme that functions to both activate and deactivate arylamine and hydrazine drugs and carcinogens[22]. The NAT2 enzyme is particularly active in the liver, gastrointestinal tract, and urinary bladder, among other organs and tissues. Due to the metabolic rate of exogenous compounds, the polymorphisms in the NAT2 gene can be classified into two types – rapid acetylator and slow acetylator. NAT2 slow acetylator is common in the Caucasian population, estimated to be around 55%. The association of the NAT2 slow acetylator with bladder risk is quite well established, serving as an outstanding example prior to the GWAS era for the replicated association between common genetic polymorphisms and complex diseases.

Appendix G: Related Work and Discussion

Identification of statistical interactions between participating factors has many practical implications. For instance, significant efforts have been made to investigate gene-environment interactions, as it is well accepted that multiple genetic and environmental factors acting in interconnected biological pathways or networks contribute to the susceptibility and progression of complex human diseases. Besides revealing the mechanisms underpinning the disease, the identification of gene-environment interactions may assist the design of targeted therapies, interventions, or preventive strategies for complex diseases. After all, the genetic variants that are most easily translated for public health or clinical utility will be those that have an obvious corresponding environmental modification identified to be capable of altering the disease risk. In contrast to the impressive accumulation of gene-environment data resources over the last decade, the progress on analytical approaches has been limited, with LR still the de facto standard[23]. LR occasionally receives critiques, albeit mainly for its lack of power or due to its excessively large required sample sizes. We have shone a light on an additional problem – lack of consistency in the presence of three common confounding effects – and characterized the practical implications (inflated type 1 error). In spite of all of these limitations, LR continues to be widely used. While the main motivations for AIM are its plausibility as a disease model and its enhanced power, consistency with respect to these

confounders gives additional ammunition motivating its use.

Various efforts have been made to enhance LR's power. One major school is based on the case-only study[23]. Under two assumptions – (1) the two risk factors are independent in the population and (2) the disease incidence rate is rare – it can be shown that LR for interaction analysis reduces to association analysis between the two risk factors in the case group only. Simulation studies have demonstrated larger power of case-only studies compared to LR. However, it was pointed out that the power gain was purely owing to these strong assumptions, since, under them, fewer parameters need to be estimated and the true interaction effect can be more easily distinguished from the null distribution with fewer degrees of freedom. Yet, in real applications, these assumptions may not hold. A gene may influence the environment to which an individual is exposed. For example, genetic makeup may be a strong determinant of lifestyle. Even for some seemingly unlikely dependency, the independence assumption can be violated indirectly, for instance, through family history. For example, a potential inheritor of the BRCA1 gene may tend to opt for oral contraceptives because of the history of breast cancer in the family. Violation of the independence assumption often leads to inflated false positive rates. Sometimes it will also result in decreased power[24]. provides such an empirical example – the case-only method missed the interaction between the ALDH2 gene and drinking status on esophageal squamous-cell carcinoma, whereas standard LR successfully detected it, because a person with the risk allele in the ALDH2 gene tends to not drink due to the flushing reaction while drinking.

New variants of the case-only method, including empirical Bayes[25] and model averaging[26], were proposed to combine the strengths of the case-only study and of LR, aiming at gaining power by exploiting the assumption of independence and yet protecting against false positives when the independence assumption is violated. However, these methods are essentially weighted averages of the case-only and case-control statistics, and hence they are necessarily liberal under the violation of the independence assumption. At the same time, case-only and its variants will be invalid for common diseases like diabetes or heart diseases due to the violation of the assumption of rare incidence. More importantly, both the case-only method and its variants share the same principle with LR, that is, that null models be multiplicative for disease risks coming from multiple factors. Therefore, the fundamental problems we discussed pertaining to LR also apply to the case-only method and its variants.

All the approaches discussed above – LR, the case-only method, and its variants – can be considered multiplicative models because their null hypotheses all posit a product of disease risks

when all risk factors are present. An alternative is the so-called additive model, which hypothesizes an additive effect of disease risks when multiple risk factors are present. This model was highly motivated by the public health goal of finding cost-effective intervention strategies for disease reduction, since departures from additive risk would identify special groups that benefit most from a given intervention. On the one hand, an interaction relevant to the public health goal is not necessarily the most biologically meaningful. On the other hand, we do realize that the additive model can be derived as a special case of AIM if we assume the phenotype we are interested in, that is, the case, has low prevalence in the population so that mathematically $\log(1-p) \approx -p$. Nonetheless, there are disease domains for which this approximation is wildly violated. One is common diseases. For instance, according to the Centers for Disease Control and Prevention (CDC), the prevalence of coronary heart disease is as high as 19.8% among persons ≥ 65 years in 2010. According to the American Diabetes Association, 11.3% of all people ≥ 20 years have diabetes in 2011. If the population is restricted to those ≥ 65 years, the prevalence reaches 26.9%. A rare disease may also become quite common when conditioned on a special population, such as Finnish heritage disease. Another important scenario is where the interest of a study is on the progression of the disease instead of its occurrence. Even though the incidence rate can be very low, the poor prognosis group, which is often considered as the 'cases', can constitute *any* fraction of the whole patient group.

Appendix H: Conclusions

We first considered the widely used LR model, identifying its limitations as a plausible model for disease risk. We further identified that LR is not theoretically supported for hypothesis testing on statistical interactions between risk factors under the following common scenarios: 1) when there are additional (unmeasured) risk factors; 2) when measured factors are "surrogates", imperfectly correlated with the true factors; 3) when there are multiple disease sub-types. Alternatively, we proposed as the null the Asymmetric Independence Model (AIM) which: i) crucially, unlike LR, is *asymmetric* with respect to "diseased" and "healthy" statuses; ii) more generally, does comport with well-accepted biological models; and iii) whose mathematical form is preserved under all of the above confounding scenarios. Most importantly, we gave a precise definition of a "synergistic" interaction, an interaction type commonly encountered in practice, for which we proved mathematically that AIM has greater detection power than LR. Experiments evaluating AIM and LR

both on four real disease case-control study domains as well as through extensive simulation studies demonstrate AIM's improved detection power over LR. Moreover, as seen in simulations, controlled experiments demonstrate both the inflated type 1 error of LR, and the type 1 error resilience and better detection power of AIM, under unmeasured, surrogate factor, and disease subtype scenarios. Through simulation studies in the Supplementary Materials, we also characterized how, for each of the two methods, power depends on an array of population variables and experiment design parameters, including sample size, effect size, case-control ratio, risk factor allele frequency, p-value threshold, main effects, correlation between risk factors, missing factors, surrogate factors, and disease subtypes. Beyond observing that AIM achieved improved power over LR under all the tested scenarios involving synergistic interactions, some of our interesting findings include that: 1) The power gain of AIM over LR is larger when the interaction effect size is small and/or when the sample size is relatively small; 2) The power gain of AIM over LR is larger when the main effect is large; 3) Fitting to its name, AIM is not only mathematically asymmetric – its power is *also* asymmetric, with respect to the case-control ratio, the allele frequency, and the log of the odds ratio; 4) While LR does have a detection power advantage over AIM for antagonistic interactions, we observed very modest power differences between the two models for the simulated antagonistic interactions we investigated, for all tested interaction effect sizes. We also note that, in many instances, it should be possible to determine the hypothesized interaction's "direction" (synergistic or antagonistic) and choose the null hypothesis model accordingly.

References

1. Hogan, M. D., Kupper, L. L., Most, B. M., & Haseman, J. K. Alternatives to Rothman's approach for assessing synergism (or antagonism) in cohort studies. *American Journal of Epidemiology*, 108:60–67 (1978).

2. Weinberg, C. R. Applicability of the simple independent action model to epidemiologic studies involving two factors and a dichotomous outcome. *American Journal of Epidemiology*, 123:162–173 (1986).

3. Novick, L. R., & Cheng, P. W. Assessing interactive causal influence. *Psychological Review*, 111:455–485 (2004).

4. Hosmer Jr, D. W., Lemeshow, S., & Sturdivant, R. X. *Applied logistic regression.* Vol. 398 (John Wiley & Sons, 2013).

5. VanderWeele, T. J., Mukherjee, B., & Chen, J. Sensitivity analysis for interactions under unmeasured confounding. *Statistics in Medicine*, 31:2552–2564 (2012).

6. Garcia-Closas, M., Thompson, W. D., & Robins, J. M. Differential misclassification and the assessment of gene–enviornment interactions in case–control studies. *American Journal of Epidemiology*, 147:426–433 (1998).

7. Verhulst, P.-F. Notice sur la loi que la population poursuit dans son accroissement. *Correspondence mathematique et physique*, 10:113-121 (1838).

8. Boyd, S., & Vandenberghe, L. *Convex Optimization.* (Cambridge University Press, 2004).

9. Wilks, S.S. The large-sample distribution of the likelihood ratio for testing composite hypotheses. *The Annals of Mathematical Statistics*, 9:60-62 (1938).

10. McClellan, J., & King, M. C. Genetic heterogeneity in human disease. *Cell*, 141.2: 210-217 (2010).

11. Knudson, A.G. Two genetic hits (more or less) to cancer. *Nature Reviews - Cancer*, 1.2: 157 (2001).

12. Phillips, P. C. Epistasis – the essential role of gene interactions in the structure and evolution of genetic systems. *Nature Review Genetics*, 9: 855-867 (2008).

13. Chen, L. et al. Comparative analysis of methods for detecting interacting loci. *BMC Genomics*, 12:344 (2011).

14. Liu, Y. et al. Genome-wide interaction-based association analysis identified multiple new susceptibility loci for common diseases. *PLoS Genetics*, 7.3: e1001338 (2011).

15. Legnani, C. et al. Venous thromboembolism in young women; role of thrombophilic mutations and oral contraceptive use. *European Heart Journal*, 23:984-990 (2002).

16. Martinelli, I. et al. Interaction between the G20210A mutation of the prothrombin gene and oral contraceptive use in deep vein thrombosis. *Arterioscler Thromb Vasc Biol*, 19:700-703 (1999).

17. Garro, A. J., & Lieber, C. S. Alcohol and cancer. *Annu Rev Pharmacol Toxicol*, 30: 219-249 (1990).

18. Castellsague, X. et al. Independent and joint effects of tobacco smoking and alcohol drinking on the risk of esophageal cancer in men and women. *International Journal of Cancer*, 82:657-664 (1999).

19. Lee, C.H. et al. Independent and combined effects of alcohol intake, tobacco smoking and betel quid chewing on the risk of esophageal cancer in Taiwan. *International Journal of Cancer*, 113:475-482 (2005).

20. Zeegers, M.P. et al. The impact of characteristics of cigarette smoking on urinary tract cancer risk: a meta-analysis of epidemiologic studies. *Cancer*, 89:630-639 (2000).

21. Gu, J. et al. Effects of N-acetyl transferase 1 and 2 polymorphisms on bladder cancer risk in Caucasians. *Mutat Res*, 581:97-104 (2005).

22. Sanderson, S., Salanti, G., & Higgins, J. Joint effects of the N-acetyltransferase 1 and 2 (NAT1 and NAT2) genes and smoking on bladder carcinogenesis: a literature-based systematic HuGE review and evidence synthesis. *Am J Epidemiol*, 166:741-751 (2007).

23. Cordell, H. J. Detecting gene-gene interactions that underlie human diseases. *Nature Reviews Genetics*, 10:392-404 (2009).

24. Wu, C. et al. Genome-wide association study identifies three new susceptibility loci for esophageal squamous-cell carcinoma in Chinese populations. *Nature Genetics*, 43:679-684 (2011).

25. Mukherjee, B., & Chatterjee, N. Exploiting Gene-Environment Independence for Analysis of Case-Control Studies: An Empirical-Bayes type Shrinkage Estimator to Trade Off between Bias and Efficiency. *Biometrics*, 64:685–694 (2008).

26. Li, D., & Conti, D. V. Detecting gene-environment interactions using a combined case-only and case-control approach. *American Journal of Epidemiology*, 169:497–504 (2009).
